# Supplementary material for: Sustainable and recyclable super engineering thermoplastic from biorenewable monomer
Source: Nat Commun. 2019 Jun 13;10:2601. doi: 10.1038/s41467-019-10582-6 (PMC6565616; doi:10.1038/s41467-019-10582-6)
Supplement: Supplementary file 1 — Supplementary Information [file 41467_2019_10582_MOESM1_ESM.pdf]

Supplementary Information for

Sustainable and recyclable super engineering thermoplastic from biorenewable monomer

Park *et al.*

1. Supplementary Tables

Supplementary Table 1. Characteristics of SUPERBIO, BPA-SEP, reported bio-based high T_g (>150 ºC) thermoplastics, commercial petrochemical SEP, and commercial bioplastics (Continue).

Polymer type	Biomass origin	Polymerization	Crystaillinity	M_W [a] (kg mol^{-1})	η_{inh} [g] (dL g^{-1})	T_g [h] (ºC)	T_{d5}/T_{d10} [i] (ºC)	Biomass content [j] (wt%)	E (GPa)	UTS (MPa)	Melt processing /recycling	Scale [k] (g)	Reference
**SUPERBIO** (Poly(arylene ether))	Sugar (isohexide)	Condensation	Amorphous	114 [b]	0.83	212	411/422	40-44	3.4	77	O	~1,000	This study
**BPA-SEP** (Poly(arylene ether sulfone))	Petrochemical (bisphenol-A)	Condensation	Amorphous	151 [b]	1.61	196	497/502	0	3.0	51	O	~100	This study
Poly(arylene ether sulfone)	Petrochemical (commercial PSU)	Condensation	Amorphous	35 [c]	0.37-0.39	190	-	0	-	-	-	-	Sigma Aldrich
Poly(arylene ether sulfone)	Petrochemical (commercial PSU)	Condensation	Amorphous	50-60 [b]	-	187	-	0	-	-	-	-	BASF
Poly(arylene ether sulfone)	Sugar (isohexide)	Condensation	Amorphous	<6 [d]	0.23-0.65	213-253	-	<40	-	-	X	~7	1-3
Poly(arylene ether ketone)	Sugar (isohexide)	Condensation	Amorphous	<7 [d]	0.29-0.31	165-170	-	44	-	-	X	~2	4
Poly(arylene ether ketone)	Sugar (FDCA)	Condensation	Amorphous	-	0.21	159	424/-	32	-	-	X	~2	5
Poly(arylene ether ketone)	Sugar (FDCA)	Condensation	Semicrystalline	>7 [e]	0.31-0.59	154-161	-/>458-470	32-42	-	-	X	~1	6
Poly(carbonate-ester)	Sugar (isohexide)	Condensation	Amorphous	32-43 [b]	0.47-0.56	167-193	>350/-	61-84	-	-	X	~35	7
Polyester	Sugar (isohexide)	Condensation	Amorphous	>6 [f]	0.13-0.67	158-209	>360/-	52	-	-	X	~6	8-10
Polyester	Sugar (isohexide/FDCA)	Condensation	Amorphous	>9 [f]	0.11-0.38	173-196	>320/-	>99	-	-	X	~9	11
Polyester	Sugar (isohexide/LA)	Condensation	Amorphous	65-180 [b]	-	150-181	385/397	52-66	-	-	X	~15	12,13
Polyester	Sugar (isohexide/SA)	Condensation	Amorphous	57 [b]	-	156	385/398	60	-	-	X	~15	14
Polyester	Sugar (glucitol)	Condensation	Amorphous	13 [b]	0.32	154	-/377	52	-	-	X	-	15

Supplementary Table 1. Characteristics of SUPERBIO, BPA-SEP, reported bio-based high T_g (>150 ºC) thermoplastics, commercial petrochemical SEP, and commercial bioplastics (Continue).

Polymer type	Biomass origin	Polymerization	Crystaillinity	M_W [a] (kg mol^{-1})	η_{inh} [g] (dL g^{-1})	T_g [h] (ºC)	T_{d5}/T_{d10} [i] (ºC)	Biomass content [j] (wt%)	E (GPa)	UTS (MPa)	Melt processing /recycling	Scale [k] (g)	Reference
Polyester	Lignin	Condensation	Semicrystalline	48 [b]	-	157	-/>305	>99	11	63	O	~8	16
			Amorphous (not melting)	-	0.83	168	-/431	>99	-	-	X	~2	17
	Terpene	Ring-opening	Amorphous	2-14 [b]	-	155-243	-/<330	<51				~0.5	18
				<15 [b]		<184	-/-	<66				~0.4	19
Polycarbonate	Sugar (isohexide)	Condensation		>14 [b]	0.37-0.80	156-175	-	31-84				~1	20, 21
	Sugar (mannose)	Ring-opening		16 [b]	-	152	-/220-240	73				~0.3	22
Polyacetal	Lignin	Condensation		36-39 [b]		152-159	265-307/-	67				~1	23,24
Polyvinylacetal		Post-modification		53 [e]		157	224/-	46				~2	25
Polyurethane	Sugar (isohexide)	Condensation		8-19 [b]	0.23-0.35	152-191	>271/-	~40				~5	26-29
Polyketone	Terpene	Radical addition		~100 [b]	-	162	-/327	>90				~1	30
Polydiene	Lignin	Metathesis		49 [b]		156	380/-	82				~0.1	31
Polyacrylate		Radical addition		18~50 [b]		154-205	302/-	~68				-	32,33
Poly(N-aromatic imide)	Itaconic acid			>19 [b]		153-238	>230/-	28-51				~1	34-37
Poly(hydroxylacrylamide)	Lactone			40-100 [b]		157-178	-	50-54				~2	38
Poly(methylene-lactide)				76-358 [b]		213-254	>305/-	>99				~0.3	39
Poly(methylene-ester)	Tulipalin A			~600 [b]		163-189	>320/-	63-72				~0.6	40

Supplementary Table 1. Characteristics of SUPERBIO, BPA-SEP, reported bio-based high T_g (>150 ºC) thermoplastics, commercial petrochemical SEP, and commercial bioplastics (Continue).

<table>
<tr><th>Polymer type</th><th>Biomass origin</th><th>Polymerization</th><th>Crystaillinity</th><th>M_W [a)] (kg mol^{-1})</th><th>η_{inh} [g)] (dL g^{-1})</th><th>T_g [h)] (ºC)</th><th>T_{d5}/T_{d10} [i)] (ºC)</th><th>Biomass content [j)] (wt%)</th><th>E (GPa)</th><th>UTS (MPa)</th><th>Melt processing /recycling</th><th>Scale [k)] (g)</th><th>Reference</th></tr>
<tr><td>Polystyrene (PS)</td><td rowspan="8">Petrochemical</td><td rowspan="2">Radical addition</td><td rowspan="3">Amorphous</td><td rowspan="10">-</td><td rowspan="10">-</td><td>100</td><td rowspan="10">-/-</td><td rowspan="8">0</td><td>2.9</td><td>32-44</td><td rowspan="10">O</td><td rowspan="10">-</td><td rowspan="10">41,42</td></tr>
<tr><td>Poly(methyl methacrylate) (PMMA)</td><td>110</td><td>3.2</td><td>71</td></tr>
<tr><td>Polycarbonate (PC)</td><td rowspan="8">Condensation</td><td>150</td><td>2.3</td><td>66</td></tr>
<tr><td>Poly(ethylene terephthalate) (PET)</td><td rowspan="3">Semicrystalline</td><td>70</td><td>3.5</td><td>60</td></tr>
<tr><td>Polyamide, Nylon 66</td><td>50-55</td><td>3.3</td><td>86</td></tr>
<tr><td>Polyether ether ketone (PEEK)</td><td>240</td><td>4.0</td><td>97</td></tr>
<tr><td>Polyphenylsulfone (PPSU)</td><td>Amorphous</td><td>220</td><td>3.6</td><td>95</td></tr>
<tr><td>Polyphenylene sulfide (PPS)</td><td rowspan="3">Semicrystalline</td><td>190</td><td>3.9</td><td>75</td></tr>
<tr><td>Poly(lactic acid) (PLA)</td><td>Lactide</td><td>60</td><td rowspan="2">100</td><td>3.5</td><td>50</td></tr>
<tr><td>Polyhydroxybutyrate (PHB)</td><td>Glucose</td><td>60</td><td>1.3</td><td>30</td></tr>
</table>

a) Weight-average molecular weight. b) Determined by GPC. c) Determined by light scattering. d) Determined by matrix-assisted laser desorption/ionization time of flight. e) Calculated based on ^{1}H NMR. f) Determined by membrane osmometry. g) Inherent viscosity. h) Glass transition temperature measured by DSC. i) 5 and 10% weight loss temperature measured by TGA. j) Weight ratio of biomass-derived matter. k) Theoretical weight of the produced polymer.

Supplementary Table 2. Characteristics of bio-based high T_g (>150 ºC) non-melt-processible polymers (pseudo-thermoplastics and thermosets).

Polymer type	Biomass origin	Polymerization	Crystaillinity	M_W $^{a)}$ (kg mol^{-1})	η_{inh} $^{c)}$ (dL g^{-1})	T_g $^{d)}$ (ºC)	T_{d5}/T_{d10} $^{e)}$ (ºC)	Biomass content $^{f)}$ (wt%)	E (GPa)	UTS (MPa)	Melt processing /recycling	Scale $^{g)}$ (g)	Reference
Polyamide (Aramid)	Sugar (FDCA)	Condensation	Amorphous (decompose when melt)	40 $^{b)}$	-	256	256/-	53	-	-	X	~10	43,44
Polyamide	Glucose		Amorphous	21-91 $^{b)}$		151-273	-/355-370	80-84	8-12.1	163-407		~3.5	45
	Itaconic acid			57-105 $^{b)}$		156-242	-/370-400	33-48	-	-		~300	46
	Lignin			-	>0.40	151-157	-	72				~1.5	47
Polyimide	Sugar (isohexide)				0.53-1.49	214-322	378-462/-	16-38		90-132		~1.5	48
Poly(ether-imide)					0.41-2.40	222-260	384-415/-	17-44		74-134		~0.5	49
Epoxy	Lignin	Epoxy			-	233 $^{h)}$	300/-	30		-		~1	50
						154-166 $^{h)}$	-/320-330	40-45				-	51
	Lignin/FDCA					176-208	-/320-350	~30				~1	52
	Lignin					150-178	229-320/-	34-84				-	53
	Lignin/FDCA					153	~320/-	69					44
	Sugar (FDCA)					152	293/-	35		84			55
	Rosin					154	311/-	38		-			56
	Lignin					168	338/-	53	5.0 $^{i)}$				57
	Phloroglucinol					177	-/306	29	-				58
	Petrochemical					120	-/-	0	2.4	67			41,42
Polyimide		Condensation	Semicrystalline			260			2.6-3.4	81-110			
Melamine formaldehyde			Amorphous			-			7	30			
Phenol formaldehyde						170			3.8	48			
Aramid (film)			Semicrystalline			-			11	155			

$^{a)}$ Weight-average molecular weight. $^{b)}$ Determined by GPC. $^{c)}$ Inherent viscosity. $^{d)}$ Glass transition temperature measured by DSC. $^{e)}$ 5 and 10% weight loss temperature measured by TGA. $^{f)}$ Weight ratio of biomass-derived matter. $^{g)}$ Theoretical weight of the produced polymer. $^{h)}$ Determined by dynamic mechanical analysis. $^{i)}$ Measured by nanoindentation test.

Supplementary Table 3. Coefficient of thermal expansion (CTE) of petrochemical plastics, silver nanowire, SUPERBIO, and BPA-SEP.

Polymer type	CTE (ppm K^{-1})	Type of materials	Melt processing /recycling	Reference
**SUPERBIO** (Poly(arylene ether))	23.8	Super engineering plastic	O	This study
**BPA-SEP** (Poly(arylene ether sulfone))	35.7			
Silver nanowire	21	Metal		41,42
PS	98	Commodity plastic		
PMMA	76			
Polycarbonate	69	Engineering plastic		
PET	70			
Nylon 66	81			
PEEK	52	Super engineering plastic		
PPSU	45			
PPS	45			
Epoxy resin	100	Thermoset	X	
Polyimide	54			
Melamine resin	60			
Phenol resin	120			

2. Supplementary Figures

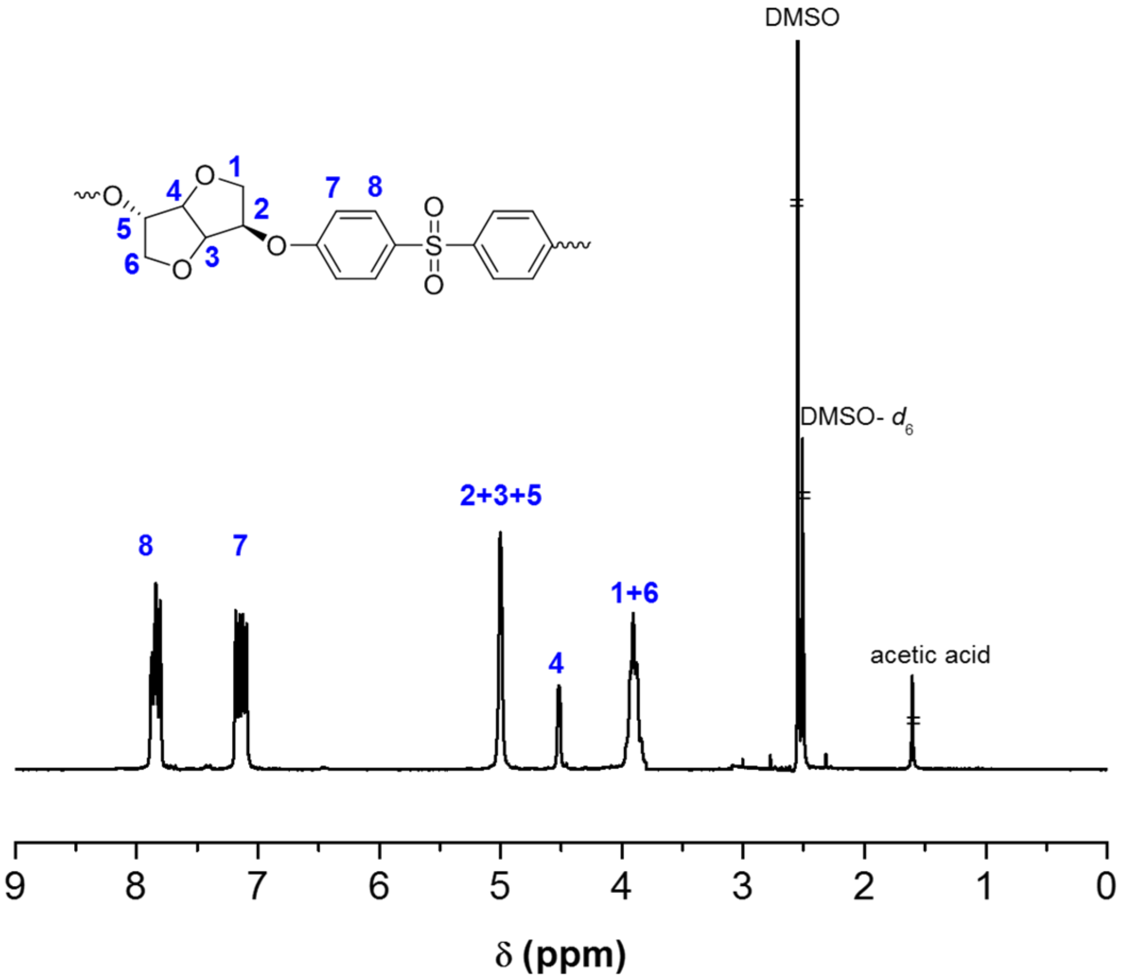

Supplementary Figure 1. ^{1}H NMR spectrum of SUPERBIO (DMSO-d_6, 300MHz).

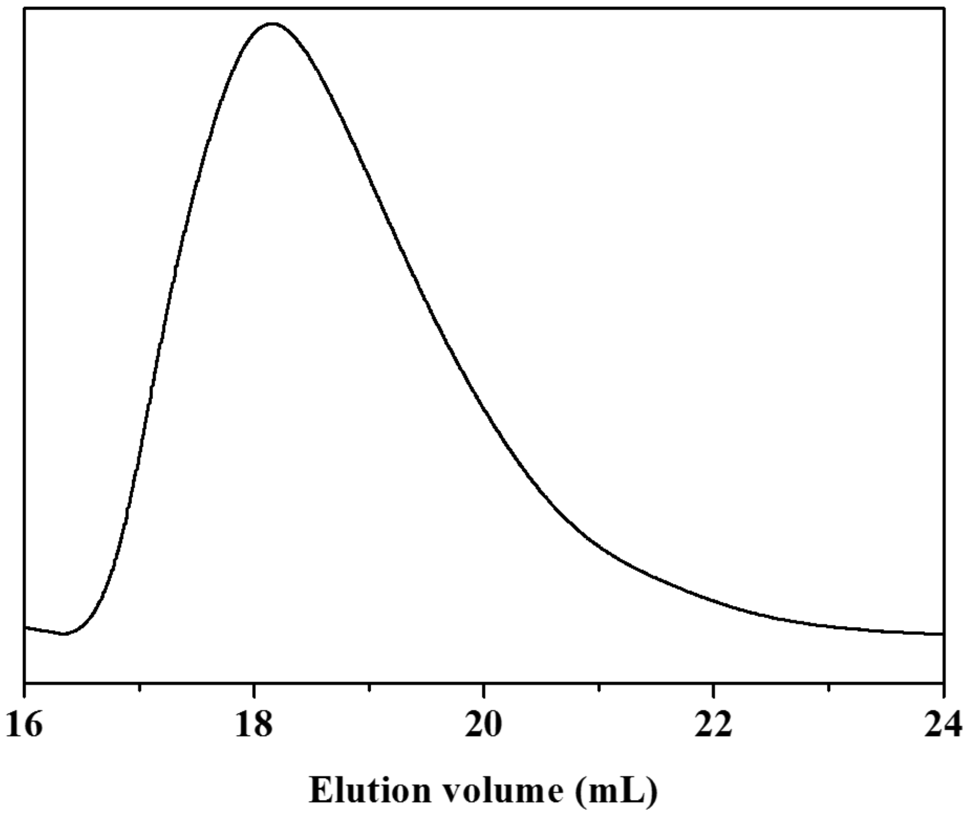

Supplementary Figure 2. DMF-GPC profile of SUPERBIO (M_n = 55.4 kg mol^{-1}, M_w = 114 kg mol^{-1}, PDI = 2.04).

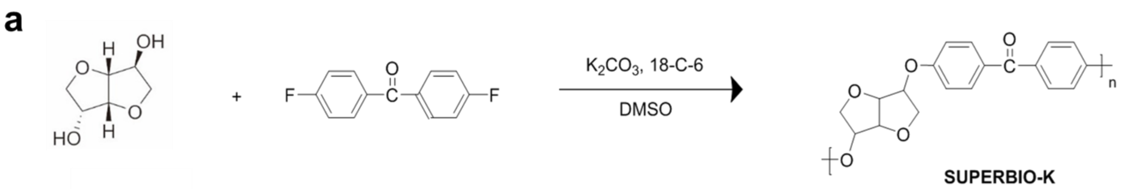

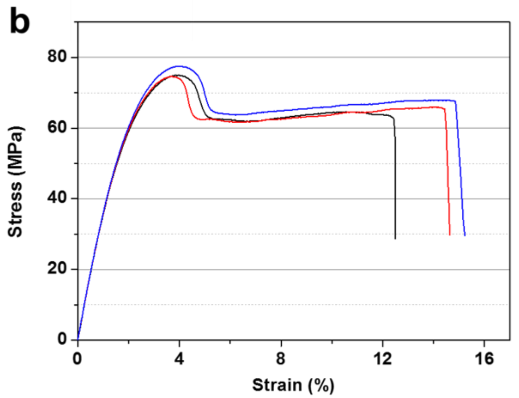

c

No.	Young's modulus (GPa)	UTS (MPa)	Elongation at break (%)	Toughness (MJ m^{-3})
1	3.9	75	11	7.5
2	3.8	75	14	8.9
3	3.7	78	14	9.6
Mean	3.8	76	13	8.7
Standard error	0.06	1.0	0.8	0.60

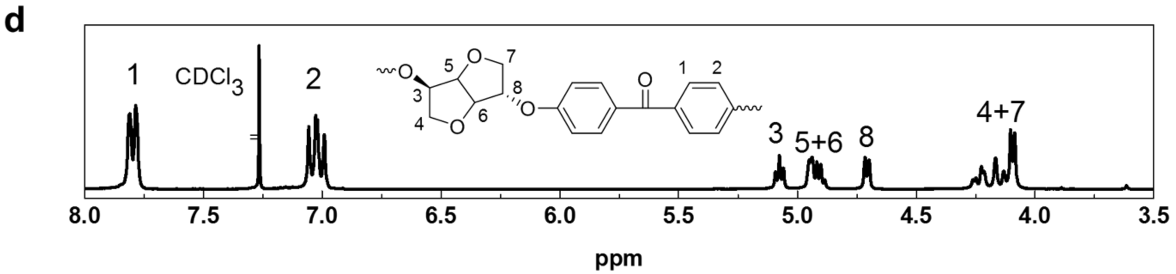

Supplementary Figure 3. (a) Synthetic route of an ISB-based ketone-type PAE called SUPERBIO-K (M_n = 44.4 kg mol^{-1}, M_w = 93.6 kg mol^{-1}, PDI = 1.98). (b) Tensile stress-strain curves, (c) data statistics (n = 3), and (d) ^{1}H-NMR spectrum of SUPERBIO-K.

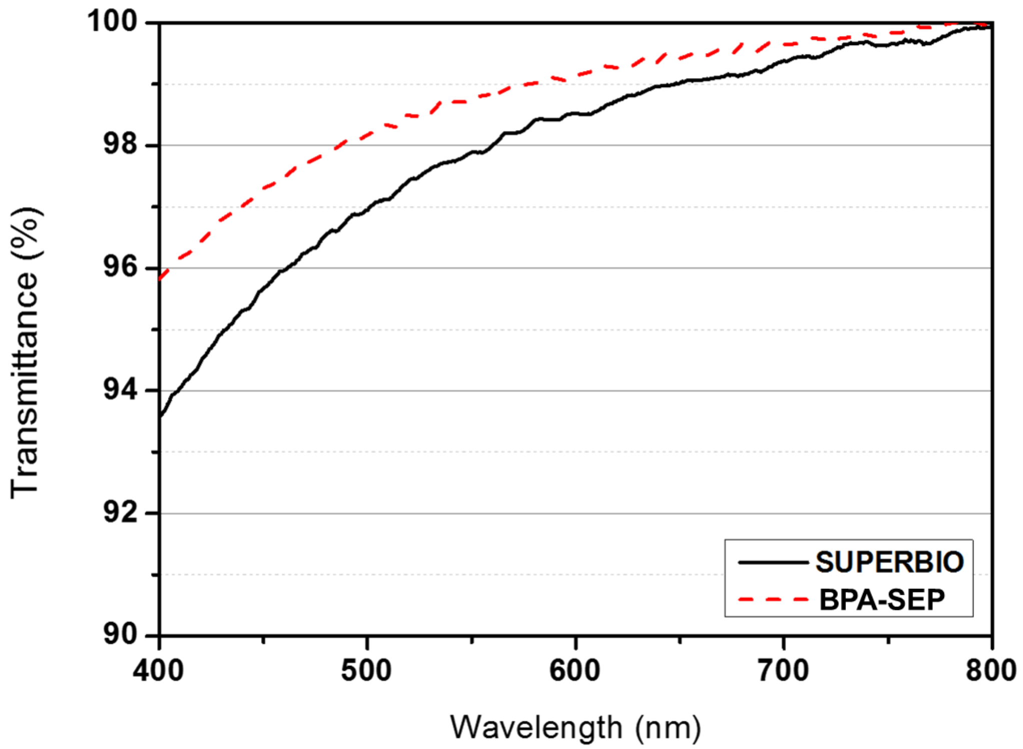

Supplementary Figure 4. UV-vis transmission spectra of films of SUPERBIO and BPA-SEP. Transparency of SUPERBIO, and BPA-SEP at 550 nm were 97.9, and 98.8%, respectively.

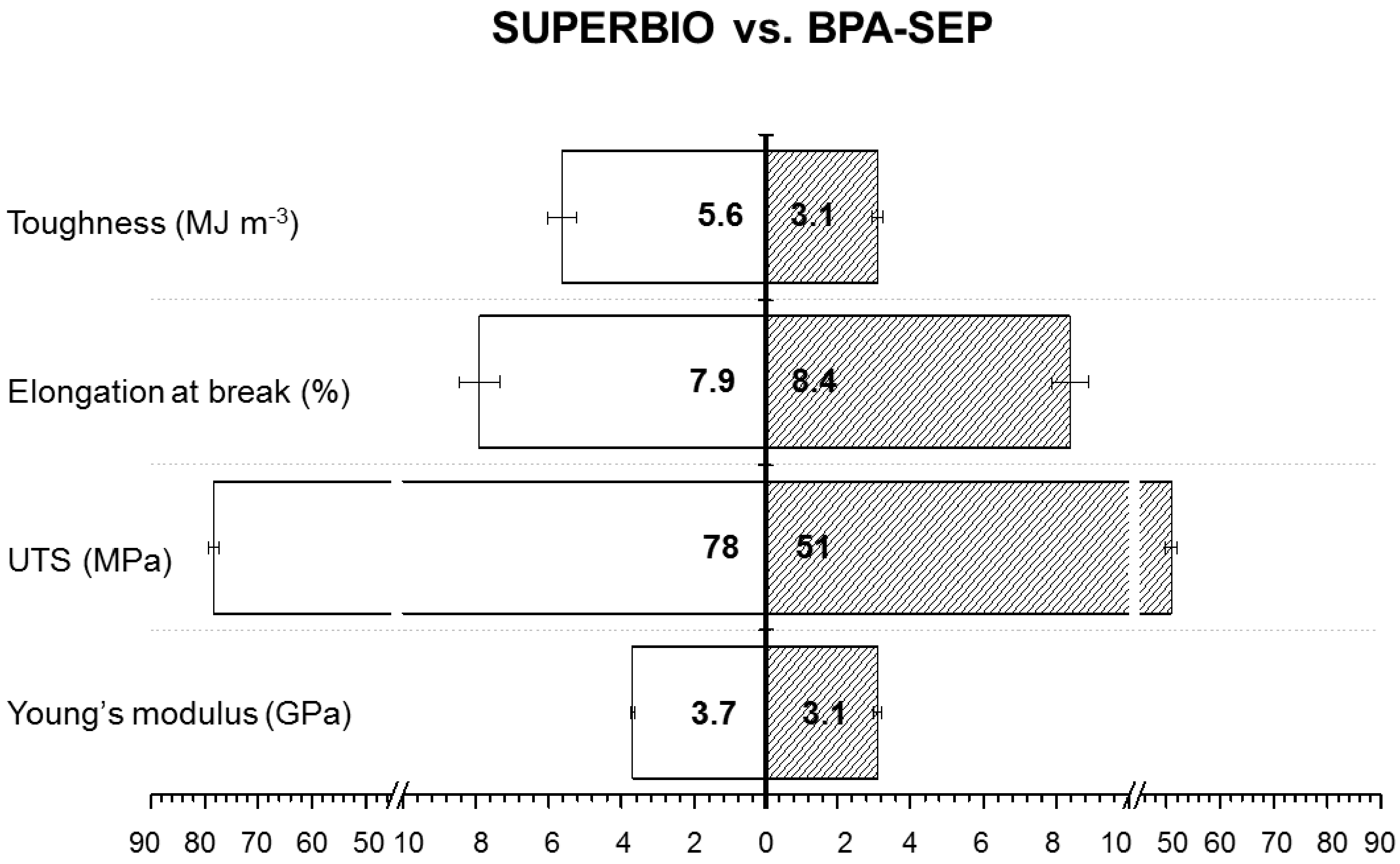

Supplementary Figure 5. Tensile properties of SUPERBIO (n = 10) and BPA-SEP (n = 8).

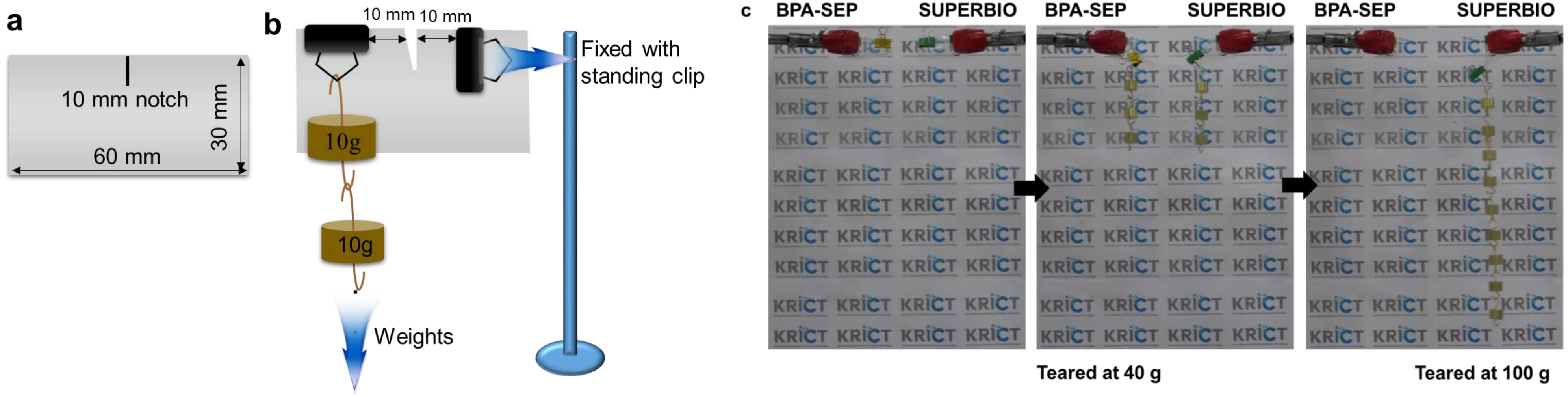

Supplementary Figure 6. A tear resistance test under applied load weights. (a) The preparation of the specimen, (b) the test method for tear resistance of specimen, and (c) snapshots of the experiment. The specimen of SUPERBIO teared at 100 g-load while BPA-SEP at 40 g-load. The detailed procedures are described in Methods section.

a

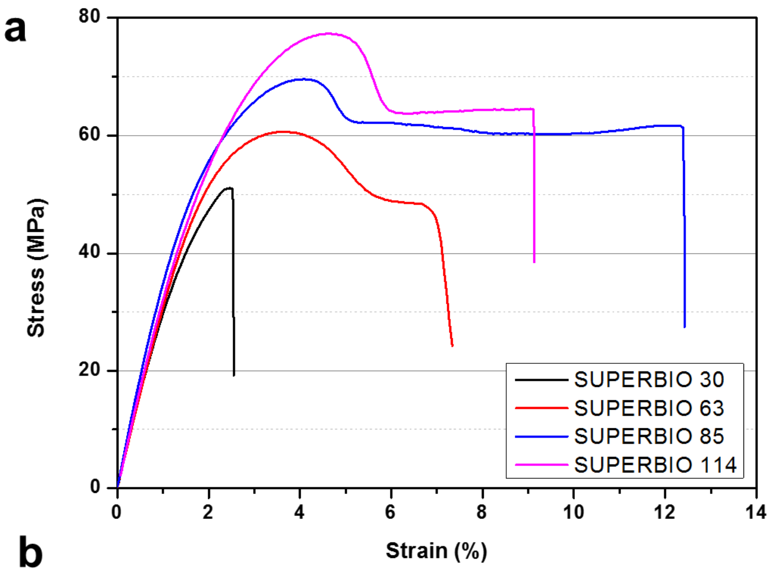

b

Code	M_w (kg mol^{-1})	Young's modulus (GPa)	UTS (MPa)	Elongation at break (%)	Toughness (MJ m^{-3})	The number of samples (n)
SUPERBIO 30	30	3.2 (± 0.03)	52 (± 1.0)	1.8 (± 0.10)	0.9 (± 0.03)	4
SUPERBIO 63	63	3.4 (± 0.09)	61 (± 1.2)	5.8 (± 0.44)	3.2 (± 0.19)	5
SUPERBIO 85	85	3.5 (± 0.13)	67 (± 1.9)	13 (± 0.4)	7.3 (± 0.31)	4
SUPERBIO 114	114	3.7 (± 0.06)	78 (± 1.0)	7.9 (± 0.6)	5.6 (± 0.42)	10

Supplementary Figure 7. (a) Representative tensile stress-strain curves and (b) data statistics of SUPERBIO with four different M_w values of 30, 63, 85, and 114 kg mol^{-1}.

a

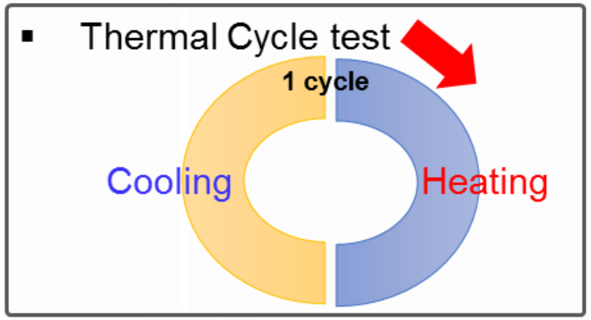

30-270 °C with a rate of 10 °C min^{-1} under N_2 atmosphere.

b

	M_w (kg mol^{-1})
Pristine	90
After 1st run	90
After 2nd run	90
After 3rd run	84
After 5th run	78

Supplementary Figure 8. The M_w changes of SUPERBIO within programmed 5 cycles of heat treatments. (a) One cycle of heat treatments consists of heating (30 to 270 ºC) and cooling (270 to 30 ºC) with a rate of 10 ºC min^{-1} under nitrogen atmosphere. (b) The M_w of SUPERBIO depending on the number of heat treatments.

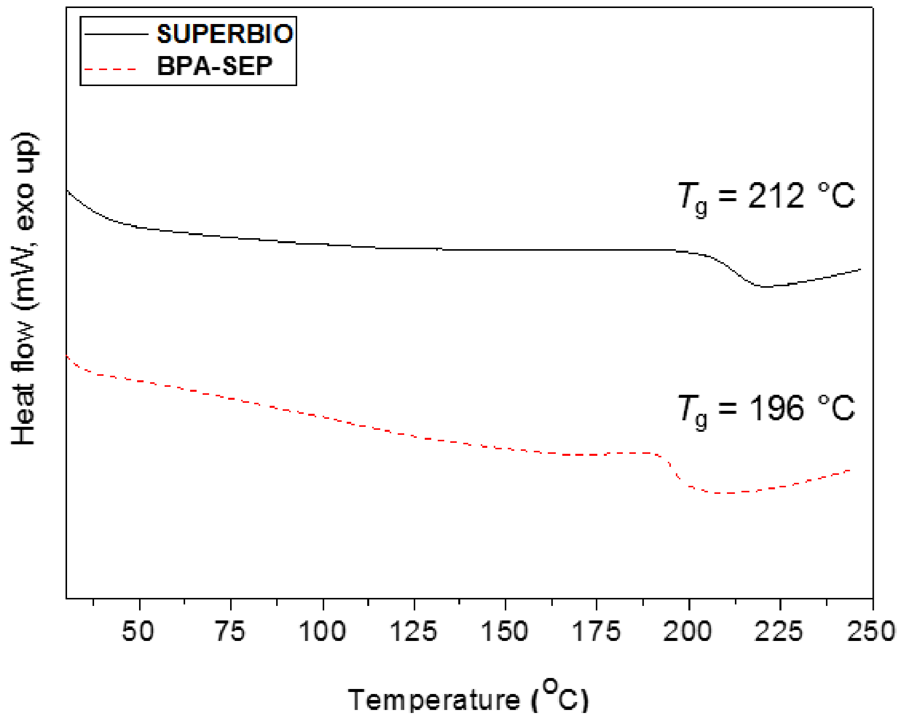

Supplementary Figure 9. DSC profiles of SUPERBIO and BPA-SEP.

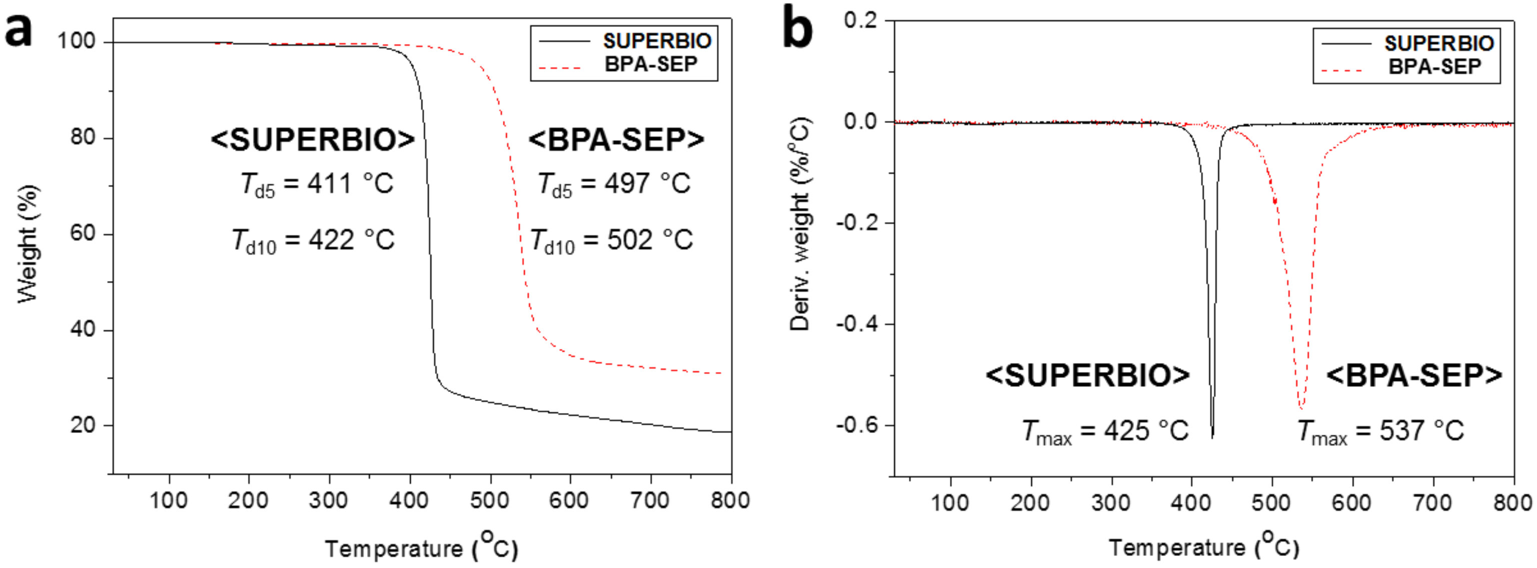

Supplementary Figure 10. (a) TGA and (b) DTG profiles of SUPERBIO and BPA-SEP.

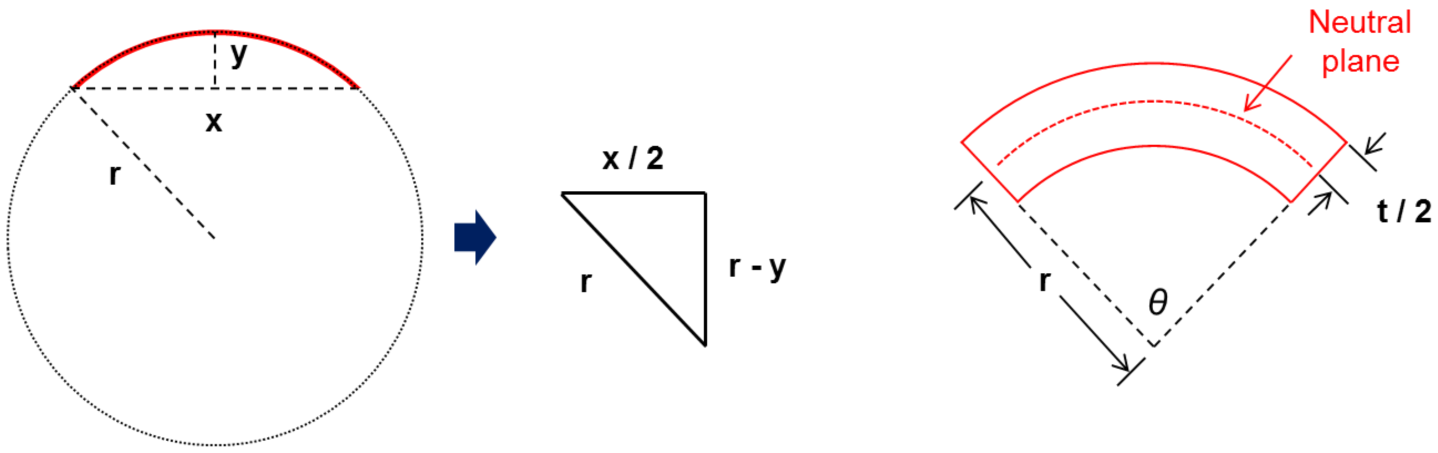

$$r^2 = (x/2)^2 + (r - y)^2$$

$$\varepsilon = \frac{\Delta l}{l} = \frac{\left(r+\frac{t}{2}\right)\theta - r\theta}{r\theta} = \frac{t}{2r}$$

$$\text{Bending radius } (r) = \frac{x^2}{8y} + \frac{y}{2}$$

$$\text{Bending strain } (\varepsilon) = \frac{t}{2r}$$

Supplementary Figure 11. Derivation of bending radius and strain from bending tests of AgNWs-coated SUPERBIO film.

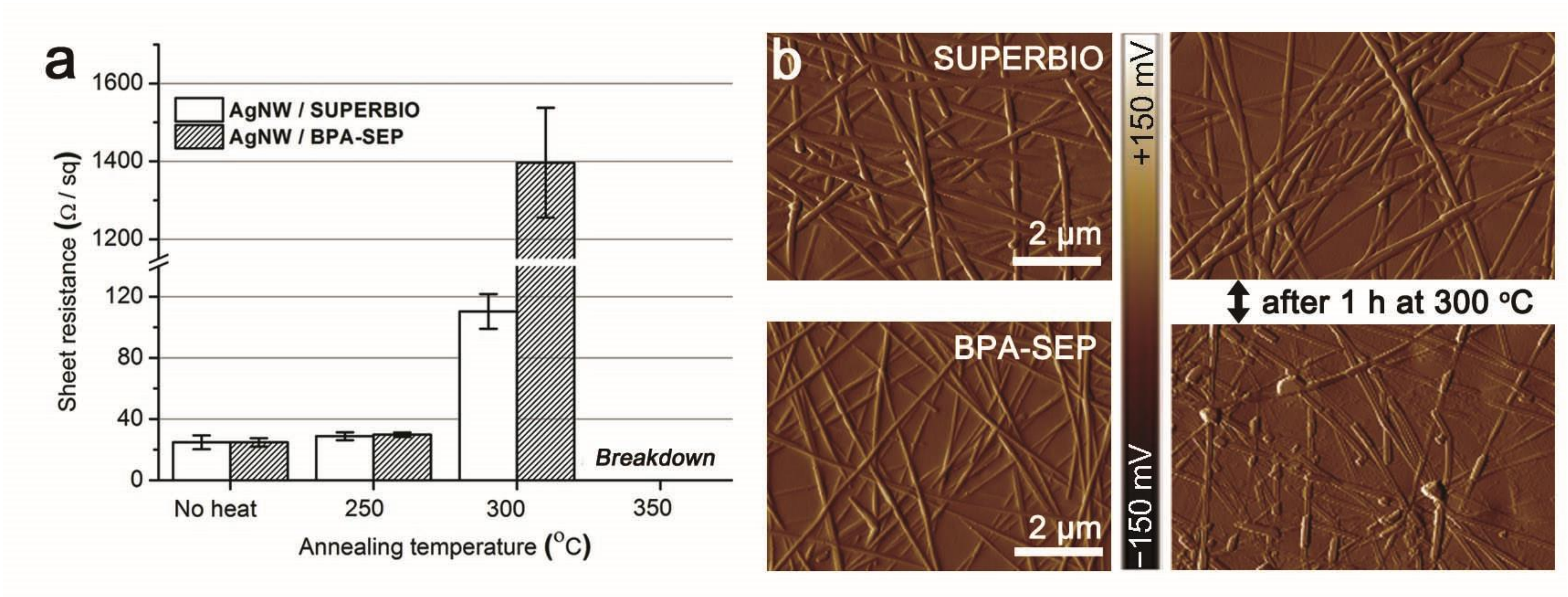

Supplementary Figure 12. (a) Sheet resistance of AgNW-coated SUPERBIO and BPA-SEP films after gradual heating to three temperature stages of 250, 300, and 350 °C, and each temperature stage was kept for 1 h under a nitrogen atmosphere. (b) Amplitude mode AFM images of AgNW-coated SUPERBIO and BPA-SEP films before and after heating at 300 °C for 1 h.

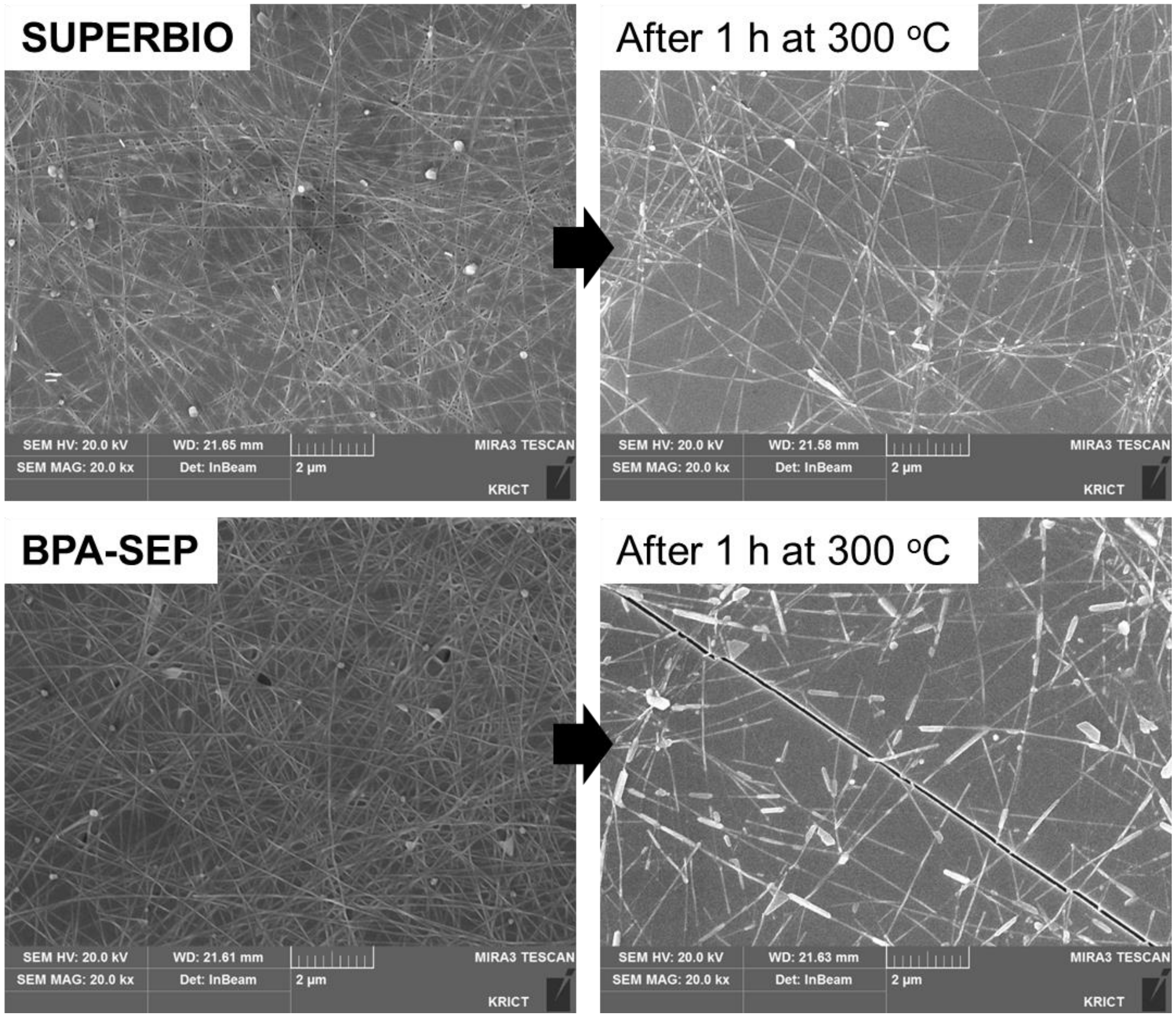

Supplementary Figure 13. FE-SEM images of AgNW-coated SUPERBIO and BPA-SEP electrodes (Left) before and (Right) after heated at 300 °C for 1 h.

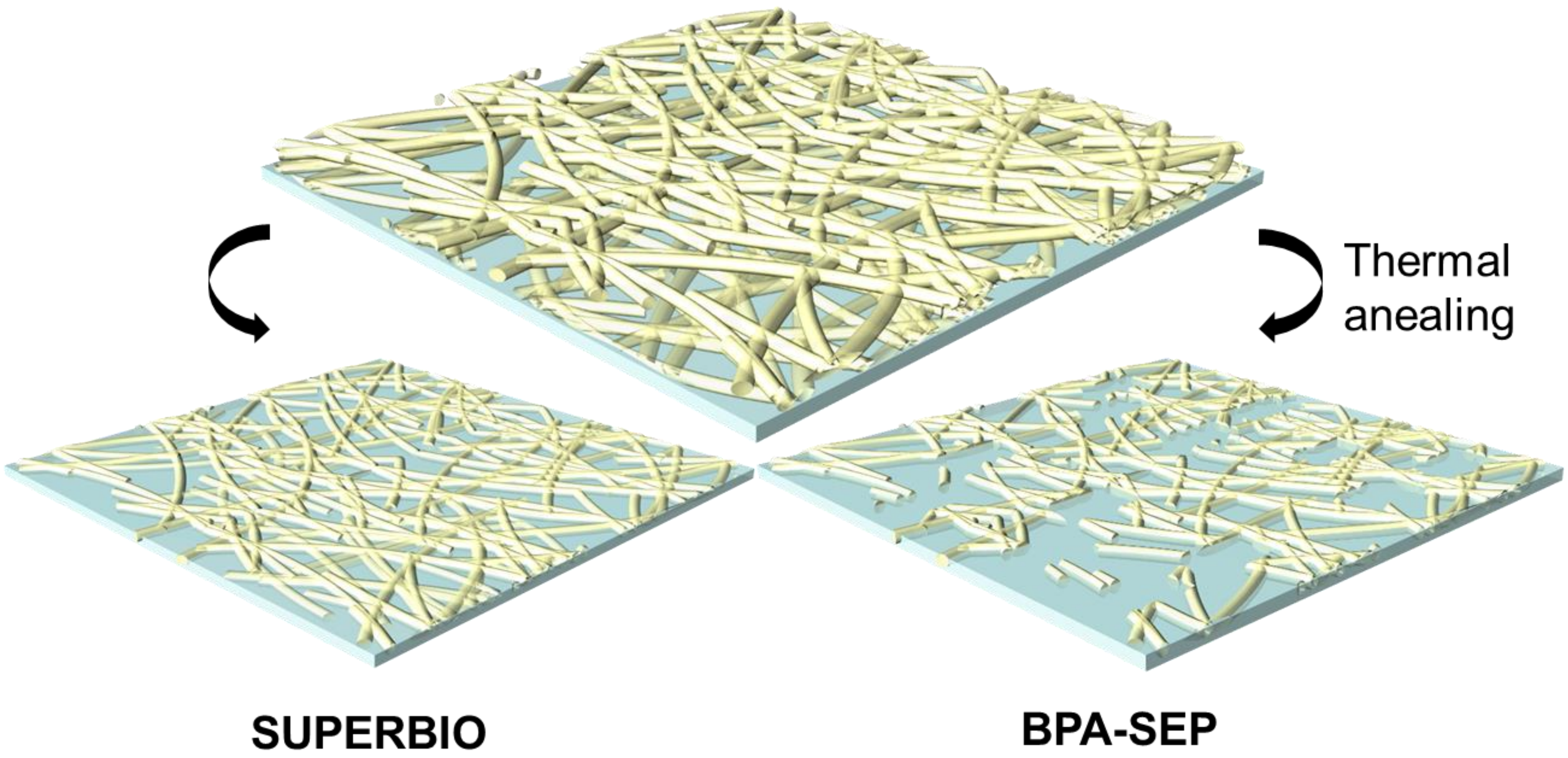

Supplementary Figure 14. Schematic illustration for the thermal stability of the AgNW percolating network on SUPERBIO and BPA-SEP films. AgNW is coated on SUPERBIO and BPA-SEP films by forming completed percolating networks.

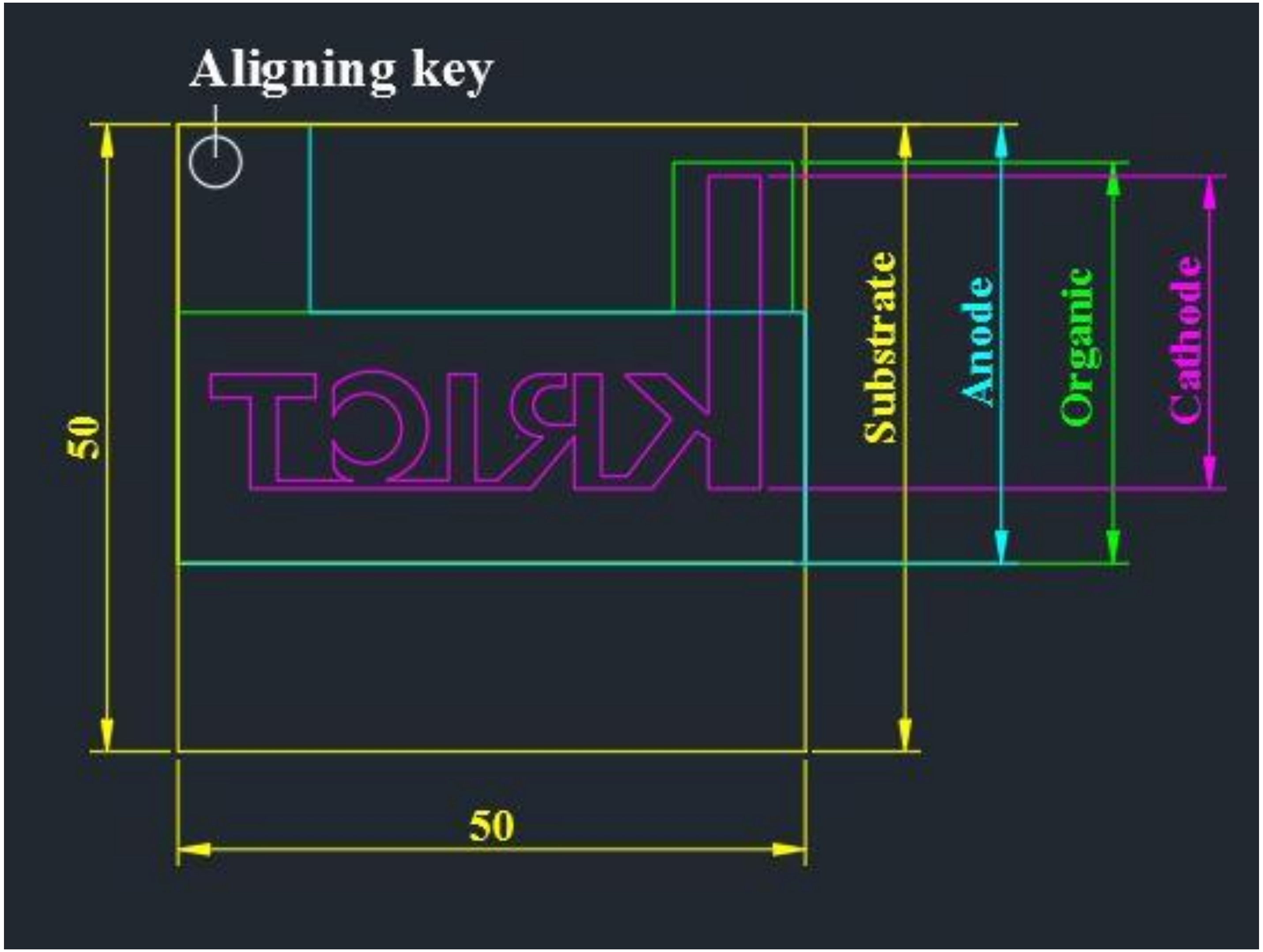

Supplementary Figure 15. OLED device layout. The OLED device was fabricated onto the 5 cm × 5 cm AgNW-embedded SUPERBIO substrate. Organic layers and cathode layers were formed in sequence onto the substrate, as illustrated in Methods section.

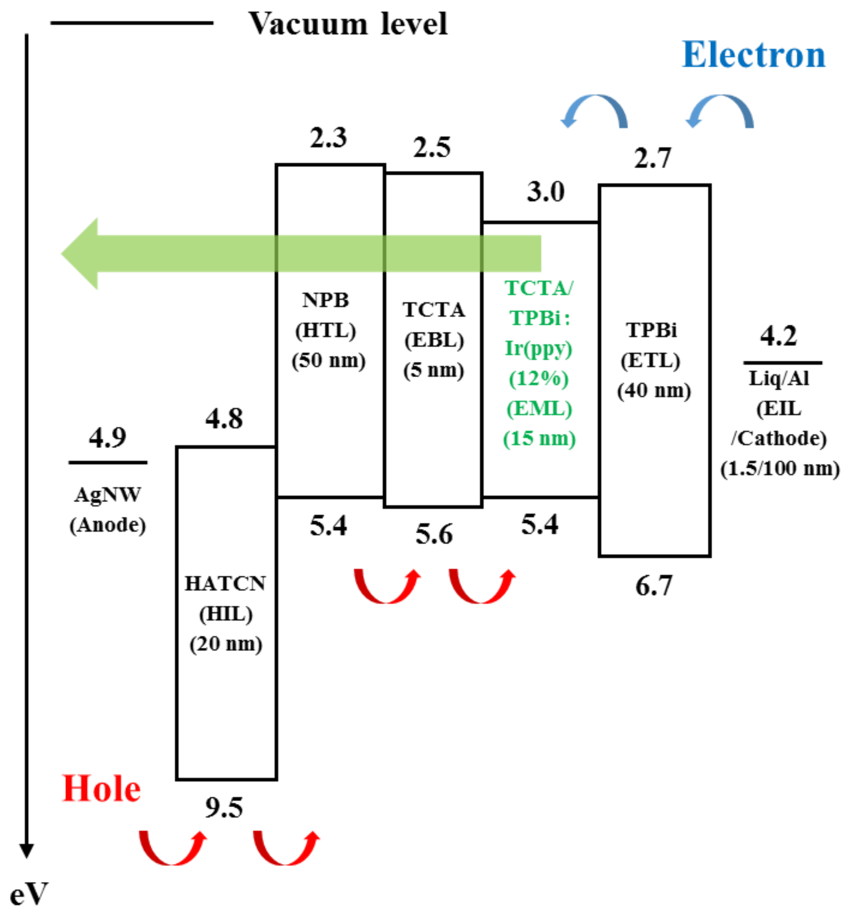

Supplementary Figure 16. The energy level diagram of an OLED device.

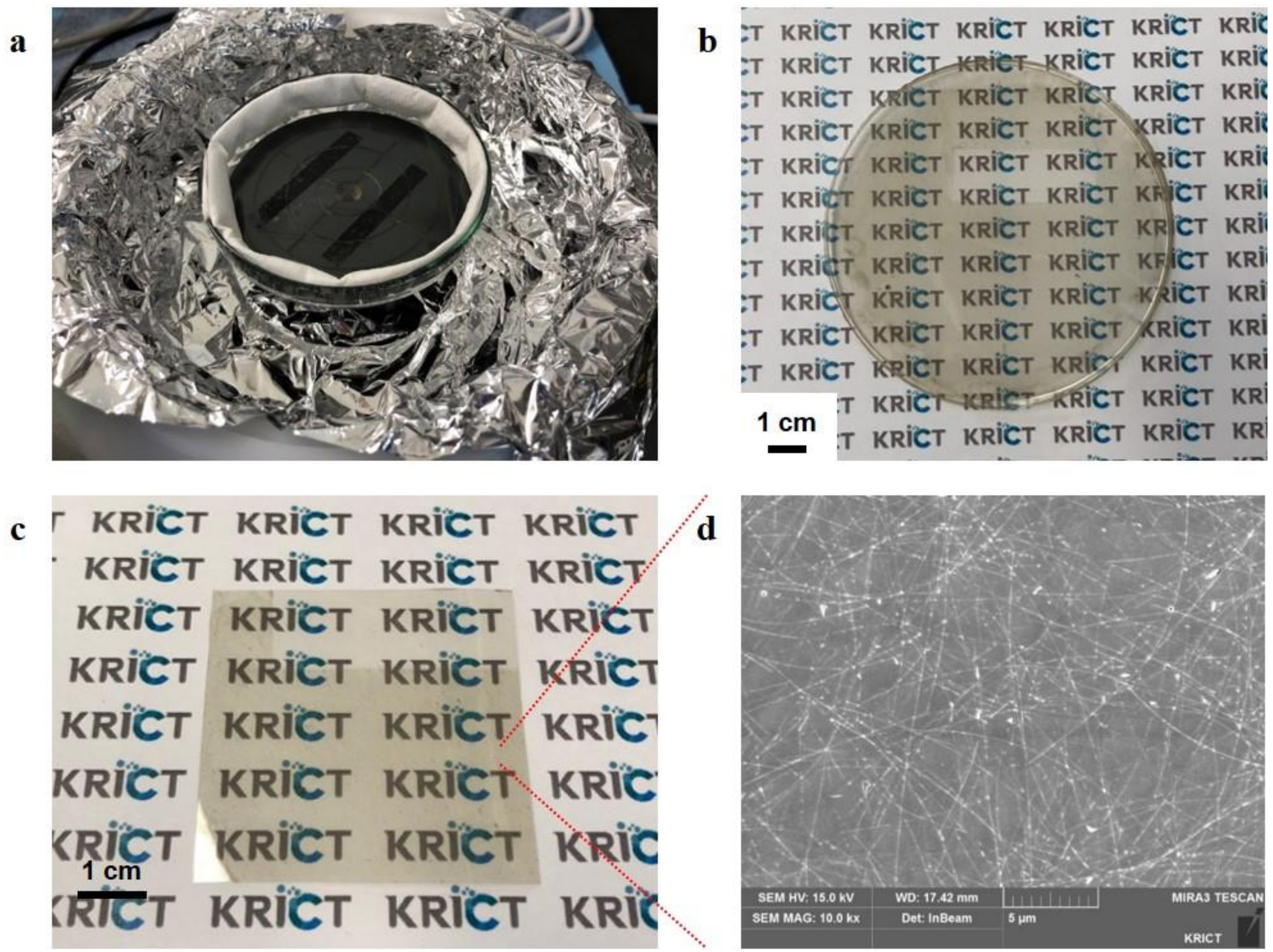

Supplementary Figure 17. ANW-embedded SUPERBIO films for OLED fabrication: (a) The set-up for AgNW spin-coating onto the glass petri dish with rectangular-shaped PDMS masking. (b) The image of AgNW-embedded SUPERBIO film in which the rectangular shape represents the bare polymeric region where AgNWs do not exist. (c) The fabricated AgNW-embedded film is cut into a 5 cm × 5 cm substrate. (d) FE-SEM image which shows the top-view of AgNW-embedded SUPERBIO film.

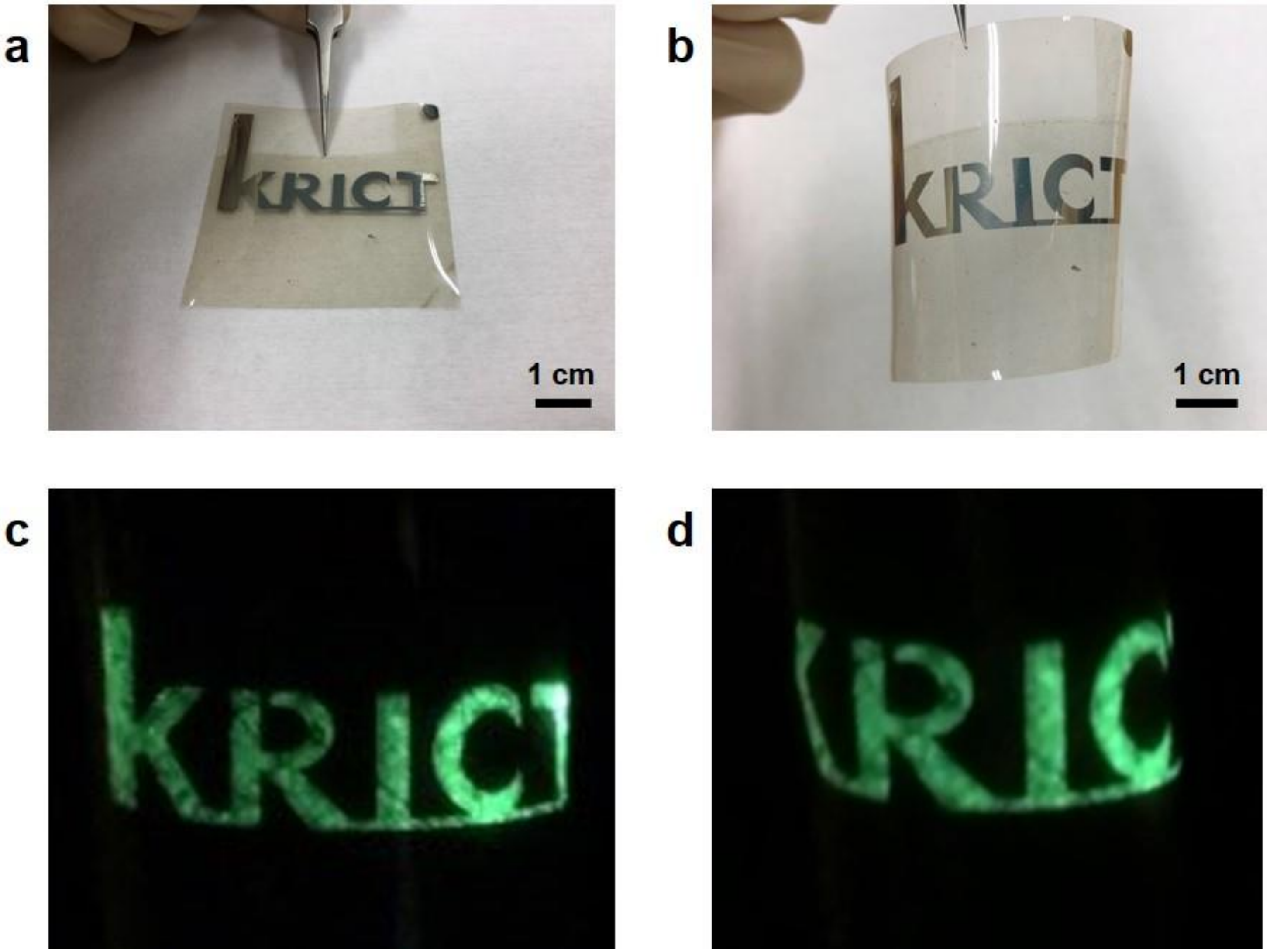

Supplementary Figure 18. The OLED device bending tests. The images of a fabricated OLED device in its flat state (a), and its bent state (b). The OLED operating tests in small bending (c), and in large bending (d).

a

SUPERBIO

Weight (mg) before/after immersion in DI water at **25** ºC for 24 h	
Before	7.9
After	7.9
M_w (g mol^{-1})	
Before	93,800
After	94,200

BPA-SEP

Weight (mg) before/after immersion in DI water at **25** ºC for 24 h	
Before	7.5
After	7.5
M_w (g mol^{-1})	
Before	137,500
After	132,400

b

SUPERBIO

Weight (mg) before/after immersion in DI water at **90** ºC for 24 h	
Before	7.3
After	7.3
M_w (g mol^{-1})	
Before	89,700
After	90,500

BPA-SEP

Weight (mg) before/after immersion in DI water at **90** ºC for 24 h	
Before	7.4
After	7.4
M_w (g mol^{-1})	
Before	134,100
After	131,800

Supplementary Figure 19. Weight (mg) and molecular weight of (Top) SUPERBIO and (Bottom) BPA-SEP before and after immersion in deionized (DI) water for 24 h at (a) 25 and (b) 90 ºC.

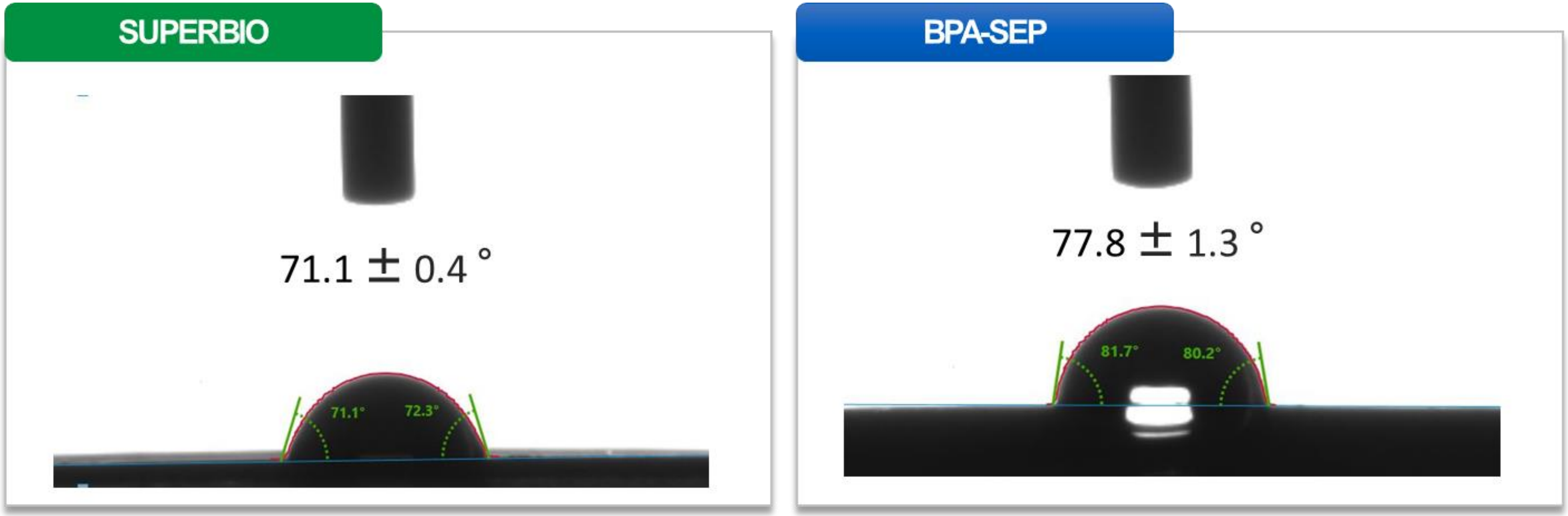

Supplementary Figure 20. Water contact angle goniometry results of SUPERBIO and BPA-SEP.

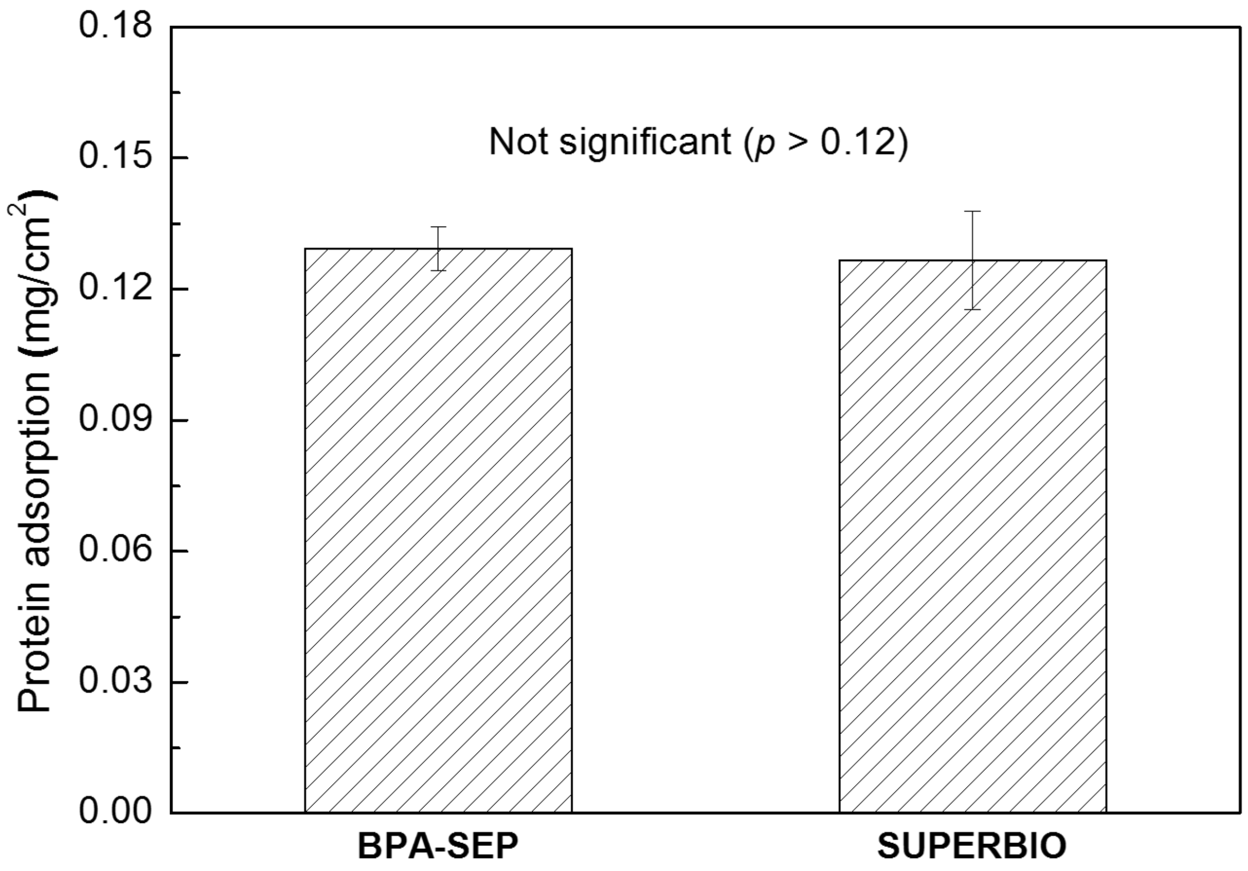

Supplementary Figure 21. Protein adsorption on SUPERBIO and BPA-SEP surfaces. Each value represents the mean and standard deviation of quintuplicate samples.

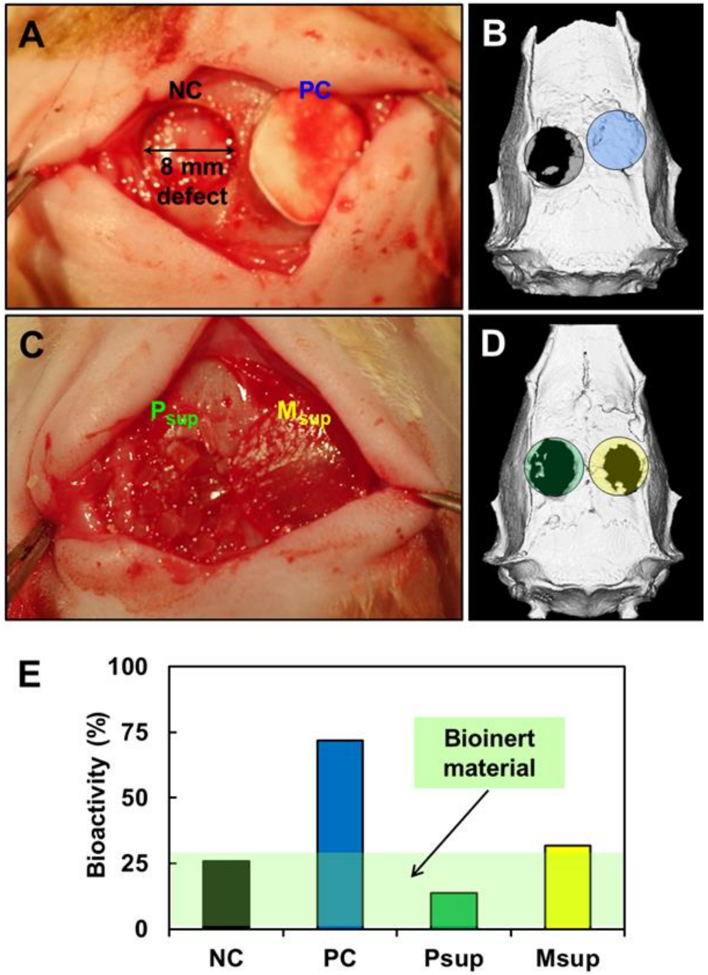

Supplementary Figure 22. (a,b) *in vivo* rat calvarial 8 mml defects: sham surgery and commercial collagen-plugged cases were negative (NC) and positive (PC) controls. (c,d) defects were plugged with SUPERBIO film (M_{sup}) and granules (P_{sup}): surgical observation and micro computed tomographic (μ-CT) images of rat calvarial defects after 12 weeks. E, relative healed bone volume (%) from μ-CT analysis.

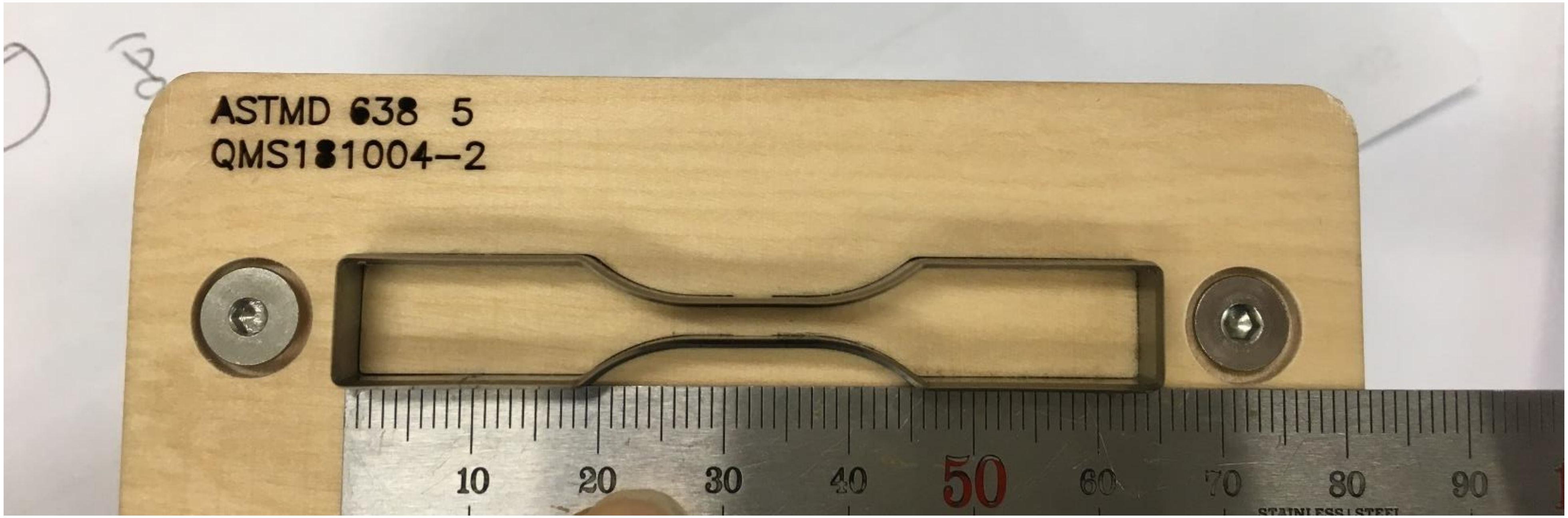

Supplementary Figure 23. A jockey type-cutter for dog-bone shape specimen following ASTM D638.

3. Supplementary Discussion

Detailed information for quantum chemical simulation.

A cause of thermal expansion is well explained with an increase in the interatomic distances.[59] The bond length increase can be understood using an anharmonic potential energy curve (PEC) with explicit vibration energy levels. All molecules have vibration energy even at zero temperature, and every bond length oscillates with respect to its expectation value of position (green circles in Fig. 3a). Upon heating, the molecules obtain thermal energy, which is converted to molecular vibration energy, and this leads to the increased population in the vibrationally excited states ($v \geq 2$). Due to the asymmetry of intermolecular potential energy, vibrational excited states have a larger expectation value of position. As marked in Fig. 3a, the expectation value of position gets away from the ground state geometry as the vibration excitation goes from $v = 1$ to $v = 4$. Therefore, the molecule in the vibrationally excited state has longer bond length compared to its ground state correspondent.

This discussion indicates that the population in the vibrationally excited states is directly related to thermal expansion: The larger vibrational energy gap implies a lower population in the vibrationally excited states. Harmonic approximation provides a relatively easy way to estimate the vibrational energy gap analytically compared to the anharmonic case. Different molecules have different interatomic potential and this yields the different shape of the PEC, especially in terms of the steepness of the potential energy wall (i.e. the second derivative of the PEC). The molecule having stronger (weaker) interatomic interaction would have a relatively steep (flat) PEC. According to a simple harmonic oscillator model, the steepness of the PEC is proportional to the square of the vibration energy gap ($\Delta E_v = E_{v=n+1} - E_{v=n}$). The molecules exhibiting a steep (flat) PEC have larger ΔE_v (smaller ΔE_v), and this results in relatively less (more) occupation in the vibrationally excited states. The relationship between the population in the vibrationally excited states and thermal expansion is discussed (vide supra). This theoretical description implies that the steepness of the PEC, significantly, is directly related to the molecular thermal expansion: The steepness of the PEC has an inverse relationship with the expansion of a single molecule. Having established the theoretical description of thermal expansion, quantum chemical simulations were conducted to describe the PEC of SUPERBIO and BPA-SEP repeating unit, and analyse and understand the origin of the single molecular expansion of the SUPERBIO polymer than the BPA-SEP polymer based on the steepness of the PEC.

Theoretical models of 'repeating units' of each polymer are selected with an assumption that the CTE value of the polymer is not significantly different from the repeating unit's value. This is a reasonable assumption since the CTE value measures the relative change. The ground state geometries are obtained with density functional theory (DFT) using the B3LYP functional with 6-31G* basis sets. Based on the ground state geometry, each bond is elongated by 0.1% up to 2.7% for the SUPERBIO repeating unit and 2.3% for the BPA-SEP repeating unit, except the bonds involving hydrogen atoms. Each geometry is re-optimized to consider the relaxation effect from angle changes. The length of the repeating unit is measured between two non-hydrogen terminal atoms (left-end oxygen atom and right-end carbon atom). The ground state geometries of the SUPERBIO and BPA-SEP repeating units are described in Fig. 3b. All quantum chemical simulations were performed using Q-Chem 4.3.[60]

Interestingly, a larger energy is required to elongate the SUPERBIO repeating unit compared with the BPA-SEP repeating unit to attain the same degree of geometric alternation (Fig. 3c). The potential energy along the relative length change of each repeating unit can be well described using a harmonic approximation within the relatively small distortion region. The second order polynomial fitting gives the coefficient of the square term, which represents the steepness of the potential wall as 94132 and 59999 for the SUPERBIO and BPA-SEP repeating units, respectively. As discussed above, this result implies that the SUPERBIO repeating unit features a larger vibration energy gap than that of the BPA-SEP repeating unit, which induces a smaller single molecular expansion value. Considering that ISB consists of only single bonds, it was expected to require less energy to force bond lengthening compared with the BPA part, which contains benzene groups. However, the simulation outcome might indicate that the ring structure of ISB induces higher geometric restrain when it is forced to have elongated bonds compared with the planar benzene group in BPA. More interestingly, the close examination of the harmonic fitting curve reveals that the steepness of SUPERBIO's PEC keeps increasing (the harmonic fitting curve tends to underestimate the last three data points). Meanwhile, the steepness of the BPA-SEP repeating unit remains relatively unchanged. The ratio of steepness between SUPERBIO and BPA-SEP repeating units is 1.41, 1.43, and 1.57, estimated with the first 5, first 10, and all data points.

Experimental, results, and discussion for Supplementary Figure 16.

The energy level of each layer is illustrated in the above diagram. The numbers illustrate the LUMO and HOMO levels for organic layers and work functions for metallic layers. The green-coloured arrow represents the direction of light emission (HATCN: 1,4,5,8,9,11-hexaazatriphenylenehexacarbonitrile as a hole-

injection layer (HIL), NPB: N,N′-di(1-naphthyl)-N,N′-diphenyl-(1,1′-biphenyl)-4,4′-diamine as a hole transport layer (HTL), TCTA: tris(4-carbazoyl-9-ylphenyl)amine as an electron blocking layer (EBL), TPBi: 2,2′,2″-(1,3,5-benzinetriyl)-tris(1-phenyl-1-H-benzimidazole) as a host, $Ir(ppy)_3$: tris[2-phenylpyridinato-C^2,N]iridium(III) as a dopant, TPBi as an electron transport layer (ETL), and Liq: 8-quinolinolato lithium as an electron injection layer (EIL)). The charge carrier transporting and green-coloured phosphorescence emitting layers were sequentially coated on the film by a thermal evaporator. Afterwards, cathode layers were formed into the letter shapes of KRICT by the thermal evaporator. Upon applying an electrical voltage of 8 V, the fabricated OLED device successfully emitted green light. Furthermore, the device maintained the light emission even when it was strongly bent.

Experimental, results, and discussion for Supplementary Figure 22.

The *in vivo* study was performed in laboratory animal center of Daegu-Gyeongbuk Medical Innovation Foundation (DGMIF, Korea) approved by the national institutional review board (IRB). Two Sprague–Dawley rats weighing 250–300 g were housed in a cage in a specific pathogen-free level-2 (SPF-2) facility; the cage was covered with soft bedding. The rats were freely accessible to food and water in a room at 23 °C and RH 50% at 8 AM and 8 PM. After a 7 d acclimation period, each rat was anesthetized with an intraperitoneal injection of 30 mg kg^{-1} of Zoletil and 10 mg $kg^{-1}$of Rompun, and the scalp was carefully incised. Each exposed calvarial was punctured to generate a critically sized defect using an 8 mm diameter trephine bur under sterile saline irrigation. The negative and positive controls were left as sham surgery and implanted of bioactive commercial collagen membrane (Bio-Gide, Geistlich, Switzerland), respectively. Whereas the experimental groups were filled with 70-μm-thick and 80-mm-long SUPERBIO film or 10 mg SUPERBIO granules (~0.5 mm in diameter). The incised periosteum and skin were closed in layers with 5-0 Vicryl sutures after the procedure. Then, the rats recovered for 12 w to confirm bioactive material-mediated tissue growth. Then, the rats were sacrificed using excessive CO_2 inhalation to harvest calvarial tissue specimens and subsequently fixed using 10% neutralized formalin until analyses. The amount of newly formed tissue through the bioactivity of each specimen was measured after anesthesia after 12 w using micro-computed tomography (μCT, SkyScan 1172; Bruker-microCT, Kontich, Belgium). A total of 683 slices were imaged (10.89 mm per slice for a 590 ms exposure) for each specimen, and the pictures were analyzed and used to generate a 3D 2000 × 1048 pixel image using CT reconstruction software (NRecon v.1.4.4; Bruker-microCT). The regenerated bone area was quantified using an image analysis program (CTAn v.1.6.0; Bruker-microCT).

We have suggested the potential application of SUPERBIO, i.e. orthodontic devices. Thus, a bio-interaction of SUPERBIO with bone was investigated by an *in vivo* bone regeneration experiments of rat calvarial defect model (Supplementary Fig. 22). As an experimental group, a 8 mm diameter defect in a rat was plugged with SUPERBIO film or granule (several mm). Sham surgery and commercial collagen-plugged cases were negative and positive controls, respectively. After 12 weeks, the surgical sites were observed, and the defect sites were visualized using an X-ray microtomography. In the experimental groups of SUPERBIO, the surgical margins of skin fully healed up, and no noticeable pus, redness, and inflammation were observed. In the positive control group, the defect site was fully filled with regenerated bone. In contrast, the SUPERBIO film group exhibited the low healed bone volume as much as that of the negative control, and the granule group gave somewhat lower healed bone volume than the other groups probably because the granules freely floated with the movement of the rat and damaged the tissue. The film and granules of SUPERBIO were peeled easily from the tissues without adhesion, and the size and shape of the film remained unchanged. This *in vivo* data indicates that SUPERBIO has bio-inertness of low interaction with tissues, which are important requirements of biomedical implants.

4. Supplementary References

1. Chatti, S., Hani, M. A., Bornhorst, K. & Kricheldorf, H. R. Poly(ether sulfone) of isosorbide, isomannide and isoidide. *High Perform. Polym.* **21**, 105–118 (2009).
2. Belgacem, C., Medimagh, R., Kricheldorf, H., Ben Romdhane, H. & Chatti, S. Copolyethersulfones of 1, 4: 3, 6-dianhydrohexitols and bisphenol A. Des. Monomers Polym. **19**, 248–255 (2016).
3. Belgacem, C., Medimagh, R., Fildier, A., Bulete, A., Kricheldorf, H., Ben Romdhane, H. & Chatti, S. Synthesis and characterization of isosorbide-based α,ω-dihydroxyethersulfone oligomers. Des. Monomers Polym. **18**, 64–72 (2015).
4. Abderrazak, H. B., Fildier, A., Romdhane, H. B., Chatti, S. & Kricheldorf, H. R. Synthesis of new poly (ether ketone) s derived from biobased diols. *Macromol. Chem. Phys.* **214**, 1423–1433 (2013).
5. Kanetaka, Y., Yamazaki, S. & Kimura, K. Preparation of poly (ether ketone) s derived from 2, 5- furandicarboxylic acid via nucleophilic aromatic substitution polymerization. *J. Polym. Sci. Part A Polym. Chem.* **54**, 3094–3101 (2016).
6. Kanetaka, Y., Yamazaki, S. & Kimura, K. Preparation of Poly (ether ketone)s derived from 2, 5-furandicarboxylic acid by polymerization in ionic liquid. *Macromolecules* **49**, 1252–1258 (2016).
7. Feng, L., Zhu, W., Zhou, W., Li, C., Zhang, D., Xiao, Y., & Zheng, L. A designed synthetic strategy toward poly(isosorbide terephthalate) copolymers: a combination of temporary modification, transesterification, cyclization and polycondensation. *Polym. Chem.* **6**, 7470–7479 (2015).
8. Reinhard, S., Matthias, R. & Matthias, B. Synthesis and properties of high-molecular-weight polyesters based on 1,4:3,6-dianhydrohexitols and terephthalic acid. *Die Makromolekulare Chemie* **194**, 53–64 (1993).
9. Kricheldorf, H. R., Behnken, G. & Sell, M. Influence of Isosorbide on Glass-Transition Temperature and Crystallinity of Poly(butylene terephthalate). *J. Macromol. Sci.* A **44**, 679–684 (2007).
10. Thiem, J. & Lüders, H. Synthesis of polyterephthalates derived from dianhydrohexitols. *Polym. Bull.* **11**, 365–369 (1984).
11. Storbeck, R. & Ballauff, M. Synthesis and properties of polyesters based on 2,5-furandicarboxylic acid and 1,4:3,6-dianhydrohexitols. *Polymer* **34**, 5003–5006 (1993).
12. Kricheldorf, H. R. & Weidner, S. M. High Tg copolyesters of lactide, isosorbide and isophthalic acid. *Eur. Polym. J.* **49**, 2293–2302 (2013).
13. Kricheldorf, H. R. & Weidner, S. M. Copolyesters of Lactide, Isosorbide, and Terephthalic Acid-Biobased, Biodegradable, High-Tg Engineering Plastics.

Macromol. Chem. Phys. **214**, 726–733 (2013).

14. Saber, C., M. Weidner, S. M., Fildier, A. & Kricheldorf., H. R. Copolyesters of isosorbide, succinic acid, and isophthalic acid: biodegradable, high Tg engineering plastics. *J. Polym. Sci. Part A Polym. Chem.* **51**, 2464–2471 (2013).
15. Japu, C., de Ilarduya, A. M., Alla, A., García-Martín, M. G., Galbis, J. A., & Muñoz-Guerra, S. Bio-based PBT copolyesters derived from D-glucose: influence of composition on properties. *Polym. Chem.* **5**, 3190–3202 (2014).
16. Kaneko, T., Thi, T. H., Shi, D. J. & Akashi, M. Environmentally degradable, high-performance thermoplastics from phenolic phytomonomers. *Nat. Mater.* **5**, 966–970 (2006).
17. Nagata, M. Synthesis, characterization, and hydrolytic degradation of copolyesters of 3-(4-hydroxyphenyl) propionic acid and p-hydroxybenzoic acid, vanilic acid, or syringic acid. *J. Appl. Polym. Sci.* **78**, 2474–2481 (2000).
18. Peña Carrodeguas, L., Martín, C. & Kleij, A. W. Semiaromatic polyesters derived from renewable terpene oxides with high glass transitions. *Macromolecules* **50**, 5337–5345 (2017).
19. Sanford, M. J., Pena Carrodeguas, L., Van Zee, N. J., Kleij, A. W. & Coates, G. W. Alternating copolymerization of propylene oxide and cyclohexene oxide with tricyclic anhydrides: access to partially renewable aliphatic polyesters with high glass transition temperatures. *Macromolecules* **49**, 6394–6400 (2016).
20. Chatti, S., Schwarz, G. & Kricheldorf, H. R. Cyclic and noncyclic polycarbonates of isosorbide (1,4:3,6-dianhydro-D-glucitol). *Macromolecules* **39**, 9064–9070 (2006).
21. Chatti, S., Kricheldorf, H. R. & Schwarz, G. Copolycarbonates of isosorbide and various diols. *J. Polym. Sci. Part A Polym. Chem.* **44**, 3616–3628 (2006).
22. Gregory, G. L., Jenisch, L. M., Charles, B., Kociok-Köhn, G. & Buchard, A. Polymers from Sugars and CO2: synthesis and polymerization of a d-mannose-based cyclic carbonate. *Macromolecules* **49**, 7165–7169 (2016).
23. Pemba, A. G., Rostagno, M., Lee, T. A. & Miller, S. A. Cyclic and spirocyclic polyacetal ethers from lignin-based aromatics. *Polym. Chem.* **5**, 3214–3221 (2014).
24. Rostagno, M., Price, E. J., Pemba, A. G., Ghiriviga, I, Abboud, K. A. & Miller, S. A. Sustainable polyacetals from erythritol and bioaromatics. *J. Appl. Polym. Sci.* **133**, 44089 (2016).
25. Rostagno, M., Shen, S., Ghiviriga, I. & Miller, S.A. Sustainable polyvinyl acetals from bioaromatic aldehydes. *Polym. Chem.* **8**, 5049–5059 (2017).
26. Lee, C.-H., Takagi, H., Okamoto, H., Kato, M., & Usuki, A. Synthesis, characterization, and properties of polyurethanes containing 1,4:3,6- dianhydro-D- sorbitol. *J. Polym. Sci. Part A Polym. Chem.* **47**, 6025–6031 (2009).

27. Beldi, M., Medimagh. R., Chatti, S., Marque, S., Prim, D., Loupy, A. & Delolme, F. Characterization of cyclic and non-cyclic poly-(ether-urethane)s bio-based sugar diols by a combination of MALDI-TOF and NMR. *Eur. Polym. J.* **43**, 3415–3433 (2007).

28. Kricheldorf, H. R., Mix, R. & Weidner, S. M. Poly(ester urethane)s derived from lactide, isosorbide, terephthalic acid, and various diisocyanates. *J. Polym. Sci. Part A Polym. Chem.* **52**, 867–875 (2014).

29. Marín, R., Alla, A., Martínez de Ilarduya, A. & Muñoz- Guerra, S. Carbohydrate-based polyurethanes: A comparative study of polymers made from isosorbide and 1,4-butanediol. *J. Appl. Polym. Sci*. **123**, 986–994 (2012).

30. Miyaji, H., Satoh, K. & Kamigaito, M. Bio- Based polyketones by selective ring- opening radical polymerization of α- pinene- derived pinocarvone. *Angew. Chem. Int. Ed.* **55**, 1372–1376 (2016).

31. Llevot, A., Grau, E., Carlotti, S., Grelier, S. & Cramail, H. ADMET polymerization of bio-based biphenyl compounds. *Polym. Chem.* **6**, 7693–7700 (2015).

32. Holmberg, A. L., Reno, K. H., Nguyen, N. A., Wool, R. P. & Epps III, T. H. Syringyl methacrylate, a hardwood lignin-based monomer for high-Tg polymeric materials. *ACS Macro Lett.* **5**, 574–578 (2016).

33. Wang, S., Bassett, A. W., Wieber, G. V., Stanzione III, J. F. & Epps III, T. H. Effect of Methoxy Substituent Position on Thermal Properties and Solvent Resistance of Lignin-Inspired Poly(dimethoxyphenyl methacrylate)s. *ACS Macro Lett.* **6**, 802–807 (2017).

34. Okada, S. & Matyjaszewski, K. Synthesis of bio- based poly (N- phenylitaconimide) by atom transfer radical polymerization. *J. Polym. Sci. Part A Polym. Chem.* **53**, 822–827 (2015).

35. Vishal, A. & Veena, C. Studies on the copolymerization of methyl methacrylate with N-(o/m/p-chlorophenyl) itaconimides. *J. Appl. Polym. Sci.* **82**, 2078–2086 (2001).

36. Vishal, A. & Veena, C. Copolymerization and thermal behavior of methyl methacrylate with N-(phenyl/p-tolyl) itaconimides. *J. Appl. Polym. Sci.* **89**, 1195–1202 (2003).

37. Satoh, K. , Lee, D. , Nagai, K. & Kamigaito, M. Precision Synthesis of Bio-Based Acrylic Thermoplastic Elastomer by RAFT Polymerization of Itaconic Acid Derivatives. *Macromol. Rapid Commun.* **35**, 161–167 (2014).

38. Britner, J. & Ritter, H. Self-activation of poly(methylenelactide) through neighboring-group effects: a sophisticated type of reactive polymer. *Macromolecules* **48**, 3516–3522 (2015).

39. Miyake, G. M., Zhang, Y. & Chen, E. Y. Polymerizability of Exo-methylene-lactide toward vinyl addition and ring opening. *J. Polym. Sci. Part A Polym. Chem.* **53**, 1523–1532 (2015).

40. Agarwal, S. & Kumar, R. Synthesis of High-Molecular-Weight Tulipalin-A-Based Polymers by Simple Mixing and Heating of Comonomers. *Macromol. Chem. Phys.* **212**, 603–612 (2011).

41. Wypych, G. Handbook of Polymers. (Elsevier Science, 2016).

42. Material Properties Database. https://www.makeitfrom.com/ (accessed September, 2018)

43. Mitiakoudis, A. & Gandini, A. Synthesis and characterization of furanic polyamides. *Macromolecules* **24**, 830–835 (1991).

44. Cureton, L. T., Napadensky, E., Annunziato, C. & La Scala, J. J. The effect of furan molecular units on the glass transition and thermal degradation temperatures of polyamides. *J. Appl. Polym. Sci.* **134**, 45514 (2017).

45. Tateyama, S., Masuo, S., Suvannasara, P., Oka, Y., Miyazato, A., Yasaki, K., Teerawatananond, T., Muangsin, N, Zhou, S., Kawasaki, Y., Zhu, L., Zhou, Z., Takaya, N & Kaneko, T. Ultrastrong, Transparent Polytruxillamides Derived from Microbial Photodimers. Macromolecules **49**, 3336–3342 (2016).

46. Ali, M. A., Tateyama, S. & Kaneko, T. Syntheses of rigid-rod but degradable biopolyamides from itaconic acid with aromatic diamines. *Polym. Deg. Stab.* **109**, 367–372 (2014).

47. Castillo, E. A., Miura, H., Hasegawa, M. & Ogawa, T. Synthesis of novel polyamides starting from ferulic acid dimer derivative. *Des. Monomers Polym.* **7**, 711–725 (2004).

48. Ji, X., Wang, Z., Yan, J. & Wang, Z. Partially bio-based polyimides from isohexide-derived diamines. *Polymer* **74**, 38–45 (2015).

49. Ji, X., Wang, Z., Wang, Z. & Yan, J. Bio-Based Poly(Ether Imide)s from Isohexide-Derived Isomeric Dianhydrides. *Polymers* **9**, 569 (2017).

50. Aouf, C., Nouailhas, H., Fache, M., Caillol, S., Boutevin, B. & Fulcrand, H. Multi-functionalization of gallic acid. Synthesis of a novel bio-based epoxy resin. *Eur. Polym. J.* **49**, 1185–1195 (2013).

51. Fache, M., Auvergne, R., Boutevin, B. & Caillol, S. New vanillin-derived diepoxy monomers for the synthesis of biobased thermosets. *Eur. Polym. J.* **67**, 527–538 (2015).

52. Fache, M., Montérémal, C., Boutevin, B. & Caillol, S. Amine hardeners and epoxy cross-linker from aromatic renewable resources. *Eur. Polym. J.* **73**, 344–362 (2015).

53. Basnet, S., Otsuka, M., Sasaki, C., Asada, C. & Nakamura, Y. Functionalization of the active ingredients of Japanese green tea (Camellia sinensis) for the synthesis of bio-based epoxy resin. *Ind. Crop. Prod.* **73**, 63–72 (2015).

54. Miao, J.-T., Yuan, L., Guan, Q., Liang, G. & Gu, A. Biobased Heat Resistant Epoxy Resin with Extremely High Biomass Content from 2, 5-Furandicarboxylic Acid and Eugenol. *ACS Sustain. Chem. Eng.* **5**, 7003–7011 (2017).

55. Deng, J., Liu, X., Li, C., Jiang, Y. & Zhu, J. Synthesis and properties of a bio-based epoxy resin from 2, 5-furandicarboxylic acid (FDCA). *RSC Adv.* **5**,

15930–15939 (2015).

56. Liu, X. & Zhang, J. High-performance biobased epoxy derived from rosin. *Polym. Int.* **59**, 607–609 (2010).

57. Wan, J., Gan, B., Li, C., Molina-Aldareguia, J., Li, Z., Wang, X. & Wang, D.-Y. A novel biobased epoxy resin with high mechanical stiffness and low flammability: synthesis, characterization and properties. *J. Mater. Chem. A* **3**, 21907–21921 (2015).

58. Ménard, R., Negrell, C., Fache, M., Ferry, L., Sonnier, R. & David, G. From a bio-based phosphorus-containing epoxy monomer to fully bio-based flame-retardant thermosets. *RSC Adv.* **5**, 70856–70867 (2015).

59. Miller, W., Smith, C. W., Mackenzie, D. S. & Evans, K. E. Negative thermal expansion: a review. *J. Mater. Sci.* **44**, 5441–5451 (2009).

60. Shao, Y. et al. Advances in methods and algorithms in a modern quantum chemistry program package. *Phys. Chem. Chem. Phys.* **8**, 3172–3191 (2006).
