# Supplementary material for: Sustainable and recyclable super engineering thermoplastic from biorenewable monomer
Source: Nat Commun. 2019 Jun 13;10:2601. doi: 10.1038/s41467-019-10582-6 (PMC6565616; doi:10.1038/s41467-019-10582-6)
Supplement: Supplementary file 2 — Description of Additional Supplementary Files [file 41467_2019_10582_MOESM2_ESM.docx]

Description of Additional Supplementary Files

**Supplementary Movie 1.** Folding and unfolding processes of SUPERBIO film.

**Supplementary Movie 2.** A tear resistance test under applied load weights.

**Supplementary Movie 3.** Thermal reliability comparison demonstrated by LEDs connected to AgNW-coated SUPERBIO and BPA-SEP electrodes on a hot plate at 300 °C.
