# Supplementary material for: Sustainable and recyclable super engineering thermoplastic from biorenewable monomer
Source: Nat Commun. 2019 Jun 13;10:2601. doi: 10.1038/s41467-019-10582-6 (PMC6565616; doi:10.1038/s41467-019-10582-6)
Supplement: Supplementary file 7 — Reporting Summary [file 41467_2019_10582_MOESM7_ESM.pdf]

nature research

Corresponding author(s): DONGYEOP OH
Last updated by author(s): Mar 1, 2019

# Reporting Summary

Nature Research wishes to improve the reproducibility of the work that we publish. This form provides structure for consistency and transparency in reporting. For further information on Nature Research policies, see Authors & Referees and the Editorial Policy Checklist.

## Statistics

For all statistical analyses, confirm that the following items are present in the figure legend, table legend, main text, or Methods section.

| n/a | Confirmed | |
|---|---|---|
| ☐ | ☒ | The exact sample size (*n*) for each experimental group/condition, given as a discrete number and unit of measurement |
| ☐ | ☒ | A statement on whether measurements were taken from distinct samples or whether the same sample was measured repeatedly |
| ☒ | ☐ | The statistical test(s) used AND whether they are one- or two-sided<br>*Only common tests should be described solely by name; describe more complex techniques in the Methods section.* |
| ☒ | ☐ | A description of all covariates tested |
| ☐ | ☒ | A description of any assumptions or corrections, such as tests of normality and adjustment for multiple comparisons |
| ☐ | ☒ | A full description of the statistical parameters including central tendency (e.g. means) or other basic estimates (e.g. regression coefficient) AND variation (e.g. standard deviation) or associated estimates of uncertainty (e.g. confidence intervals) |
| ☒ | ☐ | For null hypothesis testing, the test statistic (e.g. *F*, *t*, *r*) with confidence intervals, effect sizes, degrees of freedom and *P* value noted<br>*Give P values as exact values whenever suitable.* |
| ☒ | ☐ | For Bayesian analysis, information on the choice of priors and Markov chain Monte Carlo settings |
| ☒ | ☐ | For hierarchical and complex designs, identification of the appropriate level for tests and full reporting of outcomes |
| ☒ | ☐ | Estimates of effect sizes (e.g. Cohen's *d*, Pearson's *r*), indicating how they were calculated |

*Our web collection on statistics for biologists contains articles on many of the points above.*

## Software and code

Policy information about availability of computer code

| | |
|---|---|
| Data collection | Origin8.5 (GF3S5-3089-7904163), MS Office Pro 213 32bit (Y9NQC-FJ49B-DX87F-694JT-KTKKV) |
| Data analysis | Origin8.5 (GF3S5-3089-7904163), MS Office Pro 213 32bit (Y9NQC-FJ49B-DX87F-694JT-KTKKV) |

For manuscripts utilizing custom algorithms or software that are central to the research but not yet described in published literature, software must be made available to editors/reviewers. We strongly encourage code deposition in a community repository (e.g. GitHub). See the Nature Research guidelines for submitting code & software for further information.

## Data

Policy information about availability of data

All manuscripts must include a data availability statement. This statement should provide the following information, where applicable:

- Accession codes, unique identifiers, or web links for publicly available datasets
- A list of figures that have associated raw data
- A description of any restrictions on data availability

The data that support the findings of this study are available from the corresponding author on request.

# Field-specific reporting

Please select the one below that is the best fit for your research. If you are not sure, read the appropriate sections before making your selection.

☒ Life sciences ☐ Behavioural & social sciences ☐ Ecological, evolutionary & environmental sciences

For a reference copy of the document with all sections, see nature.com/documents/nr-reporting-summary-flat.pdf

# Life sciences study design

All studies must disclose on these points even when the disclosure is negative.

| | |
|---|---|
| Sample size | 1. in vivo experiments<br>n = 5 (the number of SD rat), SD rat weight 250-300 g<br>ISO 10993-6 Tests for local effects after implantation<br><br>2. in vitro cell line experiments. a cytotoxic effect (ISO 10993-5).<br>n = 5, (the number of wells for growing cell lines) |
| Data exclusions | No data exclusion was conducted. |
| Replication | All biological experiments were conducted once. They were not replicate. |
| Randomization | 1. in vivo<br>All rats were chosen randomly. The weight of all rats were not different from that of each in t-test (p-value > 0.05) within the whole experimental procedures.<br><br>2. in vitro cell line experiments<br>The all experiments for the negative control group and the experimental group were conducted using the same homogeneously cell containing medium. |
| Blinding | 1. in vivo<br>All in vivo experiments were conducted by a a (public) contract clinical research organization, Daegu Gyeongbuk medical innovation foundation (DGMIF) (http://www.dgmif.re.kr/eng/index.do), following ISO 10993-6:2007 Tests for local effects after implantation.<br><br>The data processing and analyses were conducted by a professional contract pathologist.<br><br>The professional contract pathologist have no information for samples and only know the sample codes.<br><br>2. in vitro cell line experiments<br>No blinding tests were conducted in the in vitro experiments. |

# Reporting for specific materials, systems and methods

We require information from authors about some types of materials, experimental systems and methods used in many studies. Here, indicate whether each material, system or method listed is relevant to your study. If you are not sure if a list item applies to your research, read the appropriate section before selecting a response.

## Materials & experimental systems

| n/a | Involved in the study | |
|---|---|---|
| ☒ | ☐ | Antibodies |
| ☐ | ☒ | Eukaryotic cell lines |
| ☒ | ☐ | Palaeontology |
| ☐ | ☒ | Animals and other organisms |
| ☒ | ☐ | Human research participants |
| ☒ | ☐ | Clinical data |

## Methods

| n/a | Involved in the study | |
|---|---|---|
| ☒ | ☐ | ChIP-seq |
| ☒ | ☐ | Flow cytometry |
| ☒ | ☐ | MRI-based neuroimaging |

## Eukaryotic cell lines

Policy information about cell lines

| | |
|---|---|
| Cell line source(s) | A fibroblast cell line L-929, from ATCC. https://www.atcc.org/products/all/CCL-1.aspx |
| Authentication | We bought the authenticated cells.<br>https://www.atcc.org/Services/Testing%20Services/Cell%20Authentication%20Testing%20Service/Cell%20Line%20Authentication%20Test%20Recommendations.aspx |
| Mycoplasma contamination | As ATCC provides, the cell lines were not contaminated by Mycoplasma. |
| Commonly misidentified lines<br>(See ICLAC register) | We don't use any commonly misidentified lines. |

## Animals and other organisms

Policy information about studies involving animals; ARRIVE guidelines recommended for reporting animal research

| | |
|---|---|
| Laboratory animals | Sprague Dawley (SD) Rat Model, Male, 9 weeks of age, |
| Wild animals | n/a The study did not involve wild animals |
| Field-collected samples | n/a The study did not involve field-collected samples |
| Ethics oversight | The in vivo biocompatibility test of SUPERBIO was conducted by a contract clinical research organization [Daegu Gyeongbuk medical innovation foundation (DGMIF)] using a rat subcutaneous model, following ISO 10993-6 “the local effects of medical devices after implantation” Annex A (Fig. 5b). The test was approved by institutional animal care and use committee (IACUC) (Korea), and the approval code is DGMIF-18012301-00. |

Note that full information on the approval of the study protocol must also be provided in the manuscript.
